# Morning light treatment for traumatic stress: The role of amygdala reactivity study protocol

**DOI:** 10.1371/journal.pone.0269502

**Published:** 2022-06-08

**Authors:** David P. Cenkner, Helen J. Burgess, Brooke Huizenga, Elizabeth R. Duval, Hyungjin Myra Kim, K. Luan Phan, Israel Liberzon, Heide Klumpp, James Abelson, Adam Horwitz, Ann Mooney, Greta B. Raglan, Alyson K. Zalta

**Affiliations:** 1 Department of Psychological Science, University of California, Irvine, California, United States of America; 2 Department of Psychiatry, University of Michigan, Ann Arbor, Michigan, United States of America; 3 Consulting for Statistics, Computing & Analytics Research, University of Michigan, Ann Arbor, Michigan, United States of America; 4 Department of Psychiatry and Behavioral Health, Ohio State University, Columbus, Ohio, United States of America; 5 Department of Psychiatry and Behavioral Sciences, Texas A&M University, Bryan, Texas, United States of America; 6 Department of Psychiatry, University of Illinois at Chicago, Chicago, Illinois, United States of America; PLoS ONE, UNITED STATES

## Abstract

**Background:**

Exposure to trauma can result in various mental health disorders including anxiety, depression, and posttraumatic stress disorder (PTSD). Although psychotherapies and pharmacotherapies exist for the treatment of these disorders, many individuals fail to receive treatment and among those who do, many remain symptomatic. Therefore, it is critical to continue developing new interventions for traumatic stress that target underlying mechanisms of pathology and offer a safe and acceptable alternative to current treatments. Morning light treatment has good potential as a novel non-invasive, low risk treatment for traumatic stress. Evidence suggests that morning light may improve traumatic stress by reducing reactivity in the amygdala, a brain region implicated in the pathophysiology of PTSD and anatomically linked to circadian photoreceptors in the eye.

**Methods:**

In this study, we aim to establish a significant dose-response relationship between duration of morning light treatment and reduction in amygdala reactivity among individuals with traumatic stress symptoms (NCT# 04117347). Using a transdiagnostic approach, sixty-six individuals with a history of a DSM-5 criterion A trauma and traumatic stress symptoms will be recruited to participate in a 5-week study. Participants will be randomized across three treatment arms based on morning light treatment duration: 15-minutes, 30-minutes, or 60-minutes of light treatment per day for four weeks. To evaluate amygdala activity, participants will undergo fMRI at pre-treatment, mid-treatment, and post-treatment. Participants will also complete clinical assessments and self-report measures of PTSD, depression, and anxiety at pre-treatment, mid-treatment, and post-treatment.

**Discussion:**

Morning light therapy may be an acceptable, feasible, and effective treatment for individuals suffering from traumatic stress. Identifying mechanistically relevant targets, and the doses needed to impact them, are critical steps in developing this new treatment approach for the sequelae of traumatic stress.

## Introduction

The mental health burden of trauma is costly for individuals and society [1]. Evidence from a national sample of U.S. adults showed that 89.7% of individuals had been exposed to a traumatic event, including 53.1% of individuals exposed to interpersonal violence [2]. Trauma contributes to a wide variety of mental health problems including posttraumatic stress disorder (PTSD), depression, anxiety, substance use, and suicidality [3, 4]. Although front-line psychotherapies and pharmacotherapies exist for traumatic stress, evidence suggests that many individuals fail to receive treatment [5, 6], remain symptomatic despite treatment [7–9], or drop out of treatment before its conclusion [10]. Furthermore, uptake of these treatments is poor for several reasons including stigma, avoidance, unpleasant side effects, and poor accessibility [11]. New treatments are needed for traumatic stress that can overcome these critical barriers to care while targeting the underlying biological mechanisms of the pathology.

Morning light is typically a device-based treatment that aims to advance circadian timing, improve sleep quality, and improve mood [12]. Research suggests that morning light therapy is efficacious for treating mood disorders such as seasonal and nonseasonal depression [13]. More recently, these improvements have also been found in individuals with PTSD. Preliminary evidence from a pilot randomized trial of individuals with probable PTSD found large improvements in PTSD symptoms (Cohen’s *d* = 0.94) for participants who underwent 4 weeks of light treatment (60 min/day) relative to participants who received a placebo treatment [14]. In a randomized controlled trial of 69 combat veterans with PTSD, 4 weeks of morning light treatment (30 min/day) led to significantly greater improvements in PTSD symptoms and rates of treatment response relative to a control treatment [15]. Kawamura and colleagues [16] explored morning light treatment as a potential augmentation of exposure-based cognitive behavioral therapy (CBT). They found that participants with PTSD and panic disorder who received morning light therapy and CBT had a significantly greater reduction of depression and anxiety symptoms when compared to participants who received sham light and exposure-based CBT; however, the sample size was quite small [16].

These findings suggest that morning light has good potential as a novel, non-invasive, low risk treatment for trauma-related disturbances in mood and arousal. While bright light is sometimes associated with some side effects (e.g., headache, eyestrain, nausea, agitation) [17], these often spontaneously remit [17, 18], and patients rarely discontinue due to side effects [18]. Bright light devices (with UV filter) are considered safe with no changes in ophthalmologic exams observed after 6 years of daily use (in fall and winter months) [19]. Further, light treatments are commercially available and can be self-administered at home, allowing easy access and dissemination once efficacy is established.

The amygdala appears to be an important transdiagnostic target for traumatic stress symptoms as it is one of the central brain regions implicated in traumatic stress [20]. The amygdala plays a critical role in emotional memory and the acquisition of fear responses [21], which are intimately related to the clinical manifestation of traumatic stress symptoms. Research has shown increased amygdala function during exposure to traumatic reminders [22, 23], and amygdala hyperactivity to salient cues has been linked to traumatic stress symptoms including symptoms of depression [24] and anxiety [25], as well as PTSD [26, 27]. In a rodent model of PTSD, inhibitory deep brain stimulation of the amygdala reduced PTSD-like behaviors, demonstrating a causal link between changes in amygdala function and PTSD-like behavior [28, 29]. This link may also occur in humans as the first published case report of deep brain stimulation targeting the amygdala in a single human showed substantial clinical improvements and reduction in PTSD symptoms [30]. These studies suggest that changes in amygdala function are causally linked to changes in PTSD-like / PTSD symptoms. Moreover, several studies have demonstrated decreased amygdala activation as a result of successful treatment of PTSD [31–33], depression [21], and anxiety disorders [34]. Thus, decreasing amygdala reactivity appears to be a potentially critical treatment target for traumatic stress.

Several converging pieces of evidence suggest that morning light may affect amygdala reactivity. The primary circadian photoreceptors in the eye are specialized cells known as intrinsically photosensitive retinal ganglion cells (ipRGCs). Preclinical research has shown that IpRGCs transmit the light signal to various brain targets, including the amygdala [12]. Additionally, previous research has found that when participants were exposed to greater light intensity, when compared to dim light, a reduction in amygdala activity was found [35]. There is only one study that has examined amygdala activity before and after a light treatment. In that study, 30 healthy male participants received a 3-week morning white light treatment (30 min/day) of varying intensity, from 100–11,000 lux. The brighter the white light, the greater the pre- to post-treatment reduction in amygdala reactivity observed during an emotional faces fMRI task [36]. Thus, there is evidence to suggest morning light treatment may reduce traumatic stress by inducing a reduction in amygdala reactivity.

Given the previous findings, morning light treatment appears to be feasible, acceptable, and capable of decreasing psychopathology across diagnoses. However, no previous studies have tested the therapeutic mechanisms of morning light treatment for traumatic stress. Here, we aim to establish a significant dose-response relationship between the duration of morning light treatment and reduction in amygdala reactivity from pre-treatment to mid-treatment and/or post-treatment using a wearable device, the Re-Timer^®^, which is commercially available. Finally, we will analyze clinician administered and self-report measures to examine changes in traumatic stress as well as changes in objective and subjective sleep measures.

## Materials and methods

### Design

The Morning Light Stress Study (MLT; HUM00161267) is an interventional clinical trial aiming to recruit 66 individuals aged between 18–60 years who have experienced a DSM-5 Criterion A trauma and meet threshold for trauma-induced hyperarousal and depression, anxiety, and stress symptoms. Community members and clinical populations with traumatic stress are being recruited.

### Participants

Participants include adults living within a 1 hour driving distance of Ann Arbor, Michigan. Recruitment methods primarily utilize social media (e.g., Craigslist and Facebook), the University of Michigan’s Health Research registry (UMHR, umhealthresearch.org), targeted emailing of University of Michigan affiliates, word of mouth, flyering in local support groups, and community gatherings. With Institutional Review Board approval, research staff also email patients who likely had traumatic stress based on diagnostic codes and clinical notes in their electronic health records by using the University of Michigan’s Electronic Medical Record Search Engine (EMERSE) [37]. Inclusion and exclusion criteria are located in Table 1.

**Table 1:** Inclusion and exclusion criteria.

**Inclusion criteria**
1. Participants between 18 and 60 years old
2. Right-handed
3. Fluent in English
4. Experienced a DSM-5 Criterion A Trauma > 1 month ago
5. Endorsing at least 2 hyperarousal symptoms at "moderately" or higher (PSS-I-5 questions 15–20) [38]
6. Endorsing a Depression Anxiety Stress Scale-21 (DASS-21) [39] score > 22
7. Physically able to travel for study visit attendance
**Exclusion criteria**
1. Significant chronic disease (e.g., uncontrolled diabetes, advanced liver disease, cancer, kidney failure, seizures)
2. Uncorrected vision problems (e.g., colorblindness, cataracts, glaucoma, retinal pathology, history of eye surgery)
3. Unstable medication use 30 days prior to or during study participation
4. Moderate or severe traumatic brain injury (i.e., loss of consciousness > 30 minutes and/or open skull or foreign object directly impacted brain tissue)
5. fMRI contraindications (e.g., ferrous-containing metal in body, claustrophobia, head or neck tattoos)
6. Lifetime psychotic or bipolar disorders
7. Current significant obsessive-compulsive disorder
8. Acute suicidality within 6 months
9. Alcohol use disorder or substance use disorder within the past 3 months
10. Diagnosed with or high risk for sleep disorder (i.e., obstructive sleep apnea, restless leg syndrome, narcolepsy)
11. Severe hearing problem
12. Intellectual disability or serious cognitive impairment
13. Photosensitizing medications
14. Medications known to reduce amygdala activation (e.g., selective serotonin reuptake inhibitors, serotonin and norepinephrine reuptake inhibitor, benzodiazepines, beta-blockers, and/or opioids)
15. Unable or unwilling to abstain from amphetamine use 24 hours prior to fMRI scans
16. Current or past 30-day psychological therapy (with exception of strictly supportive therapy)
17. Pregnant, trying to get pregnant, or breastfeeding
18. Working night shifts or other shiftwork that affects sleep
19. Previous use of light treatment device in prior 6-month period
20. Participating in another research study intervention
21. Recent (<1 month) travel outside eastern time zone
22. DSM-5 Criterion A trauma within the past month
23. High risk for winter depression during the fall/winter months (determined from presence of high self-reported seasonality and high DASS depression scores)

### Procedure

**Fig 1:**
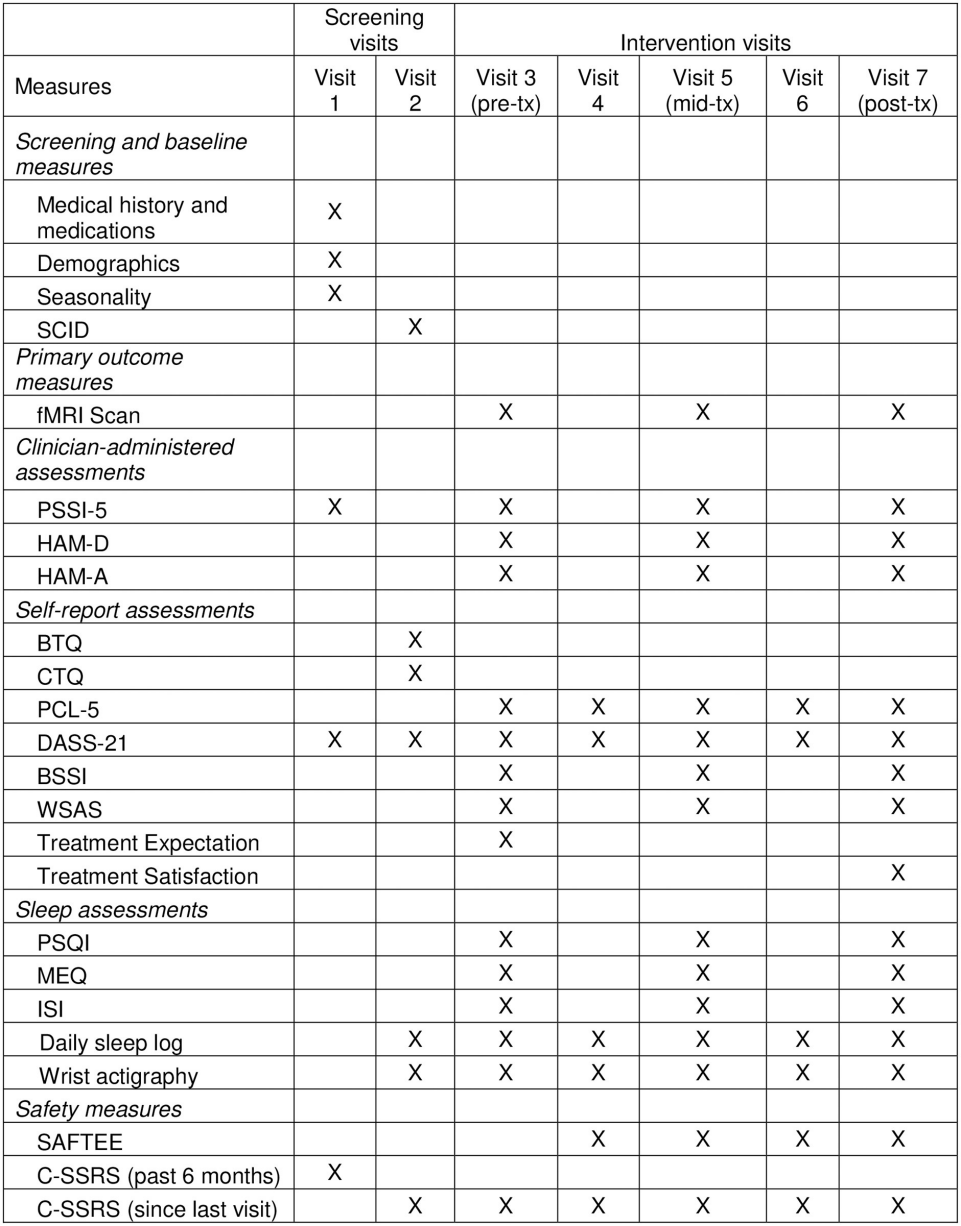
Schedule of measure administration. SCID = Structured Clinical Interview for DSM-5; PSSI-5 = PTSD Symptom Scale–Interview for DSM-5; HAM-D = Hamilton Depression Rating Scale; HAM-A = Hamilton Anxiety Rating Scale; BTQ = Brief Trauma Questionnaire; CTQ = Childhood Trauma Questionnaire; PCL-5 = PTSD Checklist for DSM-5; DASS-21 = Depression, Anxiety and Stress Scale– 21 item; BSSI = Beck Scale for Suicidal Ideation; WSAS = Work and Social Adjustment Scale; PSQI = Pittsburgh Sleep Quality Index; MEQ = Morningness Eveningness Questionnaire; ISI = Insomnia Severity Index; SAFTEE = Systematic Assessment for Treatment Emergent Events; C-SSRS = Columbia Suicide Severity Rating Scale. Wrist actigraphy is collected continuously and sleep logs are collected daily between visits 2 and 7.

Participant recruitment started in December 2019 with recruitment expected to be completed by June 2022. This is a 5-week study consisting of one week of baseline assessment and four weeks of intervention (see Fig 1 for study visit schedule). Potential participants are pre-screened for major inclusion and exclusion criteria using a combination of online survey and telephone interview. If eligible, participants then complete in-person visit 1 (eligibility visit) which includes the consenting process and collection of self-report measures related to medical history, demographics, and health information to determine further eligibility. During visit 1, participants lie in a mock fMRI scanner to ensure comfort. Final eligibility is confirmed at visit 2 (enrollment visit) after the participant meets with a Graduate- or Doctoral-level trained clinician and are interviewed using the Structured Clinical Interview for DSM-5 Disorders (SCID-5) [40] to assess for current and lifetime psychiatric diagnoses. During visit 2, participants are also administered the DASS-21 [39], the Childhood Trauma Questionnaire (CTQ) [41], and the Columbia Suicide Severity Rating Scale (C-SSRS) [42]. Finally, at visit 2, participants are given a wrist monitor (30 second epochs, Actiwatch Spectrum Plus, Respironics, Bend, Oregon) to wear on their non-dominant wrist to track their sleep throughout the study protocol. One week later, at visit 3 (pre-treatment visit), the sleep and light treatment schedule is established based on actigraphy data. Participants are assigned to follow this average sleep schedule every day for the remainder of the study. Further, during visit 3, pre-treatment outcome assessments are administered, and the pre-treatment fMRI is completed. Participants are also randomized to one of three daily light durations (15, 30, or 60 minutes/day) in a 1:1:1 ratio using a minimization approach to reduce imbalances in important covariates including combat vs. non-combat index trauma; DASS-21 [39]; use of psychoactive medication; and age, sex, and race. Morning light treatment then begins the day after visit 3.

**Fig 2:**
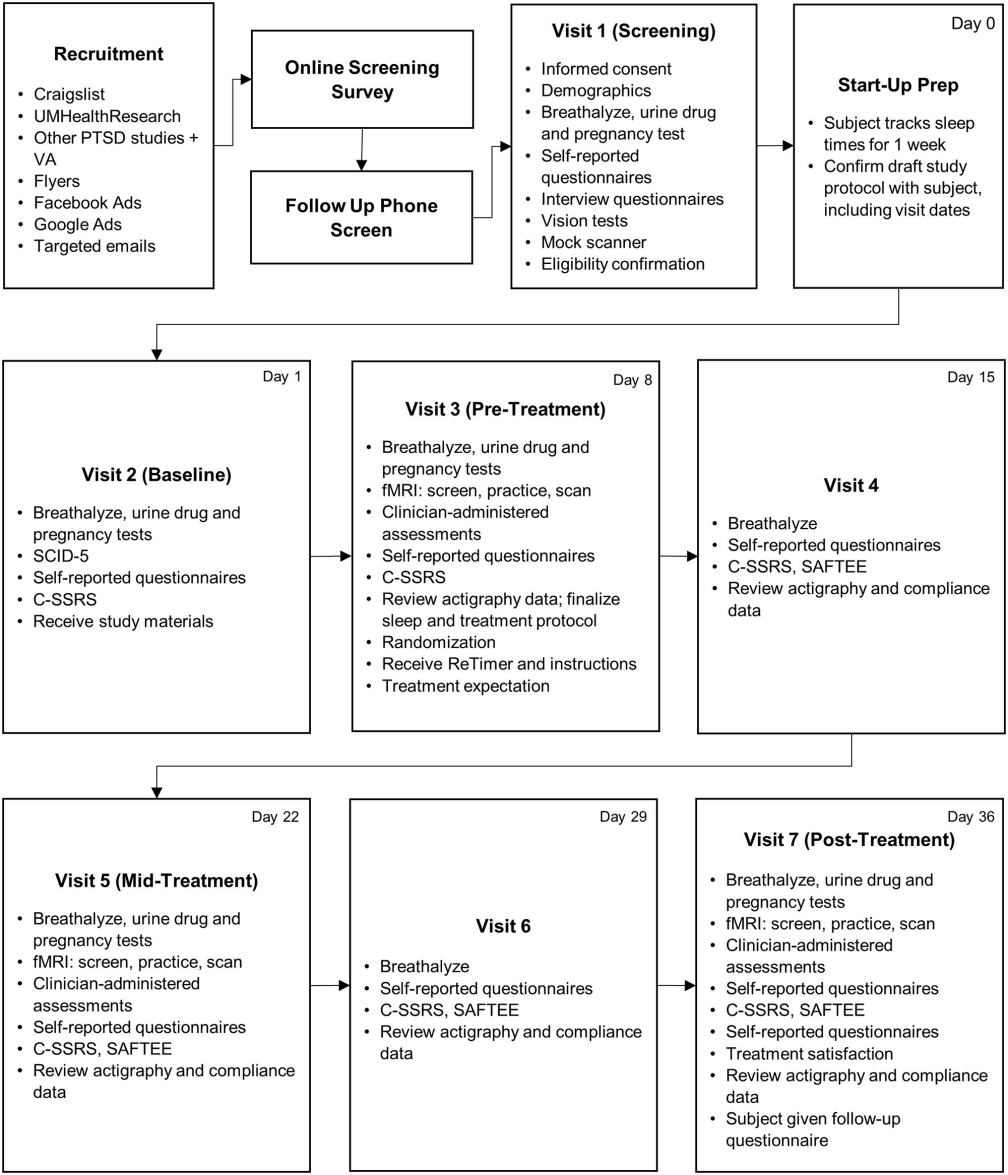
Timeline of study procedures. SCID-5 = Structured Clinical Interview for DSM-5 Disorders, C-SSRS = Columbia Suicide Severity Rating Scale, SAFTEE = Systematic Assessment for Treatment Emergent Event.

See Fig 1 for a depiction of all assessments administered at all the visits and see Fig 2 for a full timeline of study procedures. Participants are required to abstain from recreational drug use for the entirety of the study and alcohol use within 24 hours of visits. Additionally, participants are not allowed to have any caffeine one hour prior to a fMRI scan. To ensure compliance to the drug and alcohol policies, all participants are breathalyzed at the beginning of each study visit. Further, during visits 2, 3, 5, and 7 all participants are drug tested using an 8-panel urine drug test and females are administered pregnancy tests.

Note that with the outbreak of the novel Coronavirus-19 (COVID-19) in March of 2020, social distancing and protective equipment were used at in-person visits. Screening visits were modified for participant safety. For example, all screening measures were conducted virtually except for elements that required in-person assessments such as breathalyzing, drug testing, vision screening, etc. The partial virtual screening visits and informed virtual consent were conducted until July of 2021.

### Morning light treatment

The morning light treatment is self-administered at home using the Re-timer^®^ light therapy glasses. Retimer^®^ emits a green light and is designed to optimize therapeutic wavelength (~500nm, 230 μW/m^2^, 500 lux) by being close to the peak sensitivity of the circadian photoreceptors (~480 nm) [12, 43]. At visit 3 (pre-treatment), participants are randomized to a light treatment duration of 15, 30, or 60 minutes. Participants are instructed to start the light treatment the next morning immediately following their assigned wake time and are allowed to wake up one hour earlier than their average final wake time to allow time for light treatment [44]. If this occurs, the participant bedtime is also shifted earlier to avoid sleep deprivation. The earliest start time for light treatment is 6am. Participants complete light treatment sessions for 28 consecutive days at the same time and duration each day. Participants are also instructed not to sleep or meditate (closing eyes) within five hours after their assigned light treatment start time, to avoid creating a dark pulse that can counteract the effect of the light.

Adherence is assessed via light and actigraphy data measured by a monitor (30 second epochs, Actiwatch Spectrum Plus, Respironics, Bend, OR) attached to the Re-timer^®^, which is reviewed with participants at the weekly study visits. Research staff contact the participant via phone call or text 10 minutes after their assigned light treatment start time each morning to ensure that they have started the light treatment. Subjects are also given an alarm clock set to their assigned wake time and a stopwatch set to their assigned light treatment duration to promote adherence.

### Primary outcome

#### fMRI scan

The primary outcome of this study is measuring amygdala reactivity using fMRI scans. Scanning will be performed at the fMRI Laboratory at the University of Michigan on a 3.0 Tesla GE Discovery MR750 (Waukesha, WI) using a state-of-the art 32-channel radiofrequency coil and updated software (DV 26R2) with a high-performance gradient system (peak 50 mT/m, slew rate 200 T/m/s). Whole-brain blood-oxygen-level dependent (BOLD) fMRI signal measures will be acquired using a Gradient Echo sequence (TR/TE = 1200/30 ms, FOV = 21.6, slice thickness = 2.4mm) to measure brain function at rest and during task, optimized to reduce susceptibility artifact. A high-resolution T1 scan will be collected for precise anatomical localization in the same prescription as the BOLD scans to facilitate normalization. T2* images and measures of signal quality with signal-to-noise ratios (SNR) will be acquired for all participants. Only scans with high quality and scan stability with minimum motion will be analyzed.

Amygdala reactivity is measured using BOLD signal measures at each scan, which occur pre-treatment (visit 3), mid-treatment (visit 5), and post-treatment (visit 7). During each scan, participants complete the Emotional Face Assessment Task (EFAT) [45] and the Emotion Regulation Task (ERT) [46]. However, if participants find the images in the ERT too disturbing, they can opt out if desired. Prior to each fMRI scan, participants go through practice runs of the EFAT and ERT with research staff to ensure they understand the task. At the beginning of each MRI session, a resting state scan measure is captured for each participant before initiating the EFAT task.

#### Emotional Face Assessment Task (EFAT)

The EFAT has previously been shown as an effective measure of amygdala reactivity [45]. Participants view a set of three faces and are asked to match one facial expression at the top of the screen with the closest facial expression of the other two at the bottom of the screen. The pictured faces show one of five facial expressions: anger, fear, happiness, sadness, and neutrality. The matching expression also shows one of those five expressions while the incorrect match has a neutral facial expression. The EFAT uses a block design with blocks based on target face expression (4 trials per block, each trial 5 seconds, each block 20 seconds). There are 9 face blocks per run with 3 blocks of each face emotion type, presented in pseudorandom order. Nine baseline blocks are interleaved with the face blocks, consisting of shape (circle, rectangle, triangle) matching trials. Participants complete 2 runs of this task and each run lasts 6 minutes and 12 seconds. Amygdala reactivity in response to each type of facial expression is assessed by fMRI; consideration will be given specifically to amygdala reactivity to negative faces. In addition to the measure of amygdala reactivity, we also measure accuracy and response time to verify participants understand the task, are paying attention, and following directions.

#### Emotion Regulation Task (ERT)

The ERT is designed to measure a person’s ability to regulate their emotions. Participants are shown a series of either negative (e.g., violence) or neutral (e.g., light switch) images from the International Affective Picture System (IAPS) [47]. They are instructed to increase their emotional response and ruminate on negative images (“maintain”), decrease their emotional response by rationalizing negative images (“reappraise”), or simply view neutral images without any meaningful interpretation (“look”). The ERT uses a block design; blocks are based on the viewing instructions (reappraise, maintain, look), presented in a pseudorandom order. Each block lasts 20 seconds (4 pictures presented for 5 seconds in each block), and each block is presented twice per run. Baseline blocks, consisting of a fixation cross for 20 seconds, are interleaved with the reappraise, maintain, and look blocks, for a total of 12 blocks per run. A prompt is provided before each block (5 seconds) to indicate whether participants should engage in the “maintain,” “reappraise,” or “look” instructions for the block. Maintain and reappraise instructions appear prior to blocks of negative images while look instructions appear for blocks of neural images. After each block, participants are asked to rate “How negative do you feel?” from 1 (not at all) to 5 (extremely). Participants complete 4 runs of this task, each run lasts 5 minutes and 10 seconds. Amygdala reactivity in response to each type of block is assessed by fMRI; consideration will be given specifically to amygdala reactivity during maintain blocks. In addition to the measure of amygdala reactivity, we also measure self-reported affect after each block.

#### Resting-state

Resting state is designed to probe intrinsic connectivity patterns at rest. Participants were instructed to fixate on a crosshair on a blank gray screen, relax, and let their mind wander without falling asleep for 8 minutes.

### Clinician-administered assessments

Participants also meet with a clinician at pre-treatment (visit 3), mid-treatment (visit 5), and post-treatment (visit 7) to assess for current traumatic stress symptoms. These assessments include the Posttraumatic Stress Disorder Symptom Scale Interview for DSM-5 (PSSI-5) [38], Hamilton Depression Rating Scale (HAM-D) [48], and Hamilton Anxiety Rating Scale (HAM-A) [49]. Clinical assessment interviews are video recorded, at the participant’s discretion, and 20% of tapes will be assessed for fidelity by the Principal Investigator (PI, AKZ). All assessors, including the PI (AKZ), are blind to treatment condition.

### Self-reported clinical assessments

Participants complete self-report measures of past traumatic stress during their pre-treatment. These include the Brief Trauma Questionnaire (BTQ) [50] and Childhood Trauma Questionnaire (CTQ) [41].

At all weekly visits, participants report current traumatic stress symptoms on the PTSD Checklist for DSM-5 (PCL-5) [51] and Depression, Anxiety and Stress Scale– 21 item (DASS-21) [39]. Additional clinical and functioning measures are assessed at pre-treatment, mid-treatment, and post-treatment including the Beck Scale for Suicidal Ideation (BSSI) [52] and Work and Social Adjustment Scale (WSAS) [53].

After research staff have demonstrated the hands-on use of the Re-timer^®^ to participants, but before they begin the light treatment itself, participants fill out a Treatment Expectation Questionnaire which measures the participant’s expectations of the effectiveness of the treatment. At the post-treatment assessment, participants fill out a Treatment Satisfaction Questionnaire that assesses both the participant’s feelings of the treatment effectiveness and the participant’s satisfaction with the treatment. At study completion, participants are also given an optional follow-up questionnaire assessing compliance and feedback to be mailed back anonymously.

### Sleep assessments

Sleep assessments include both objective measures (i.e., wrist actigraphy) and subjective measures (i.e., self-reported ratings). Objective sleep measures include wrist actigraphy collected by wearing a Philips Actiwatch Spectrum Plus (Respironics, Bend, OR) daily during the study starting at their pre-treatment. To supplement this, participants also fill out three daily logs. The first assesses daily bed and wake times. The second assesses daily alcohol, caffeine, and medication consumption. The third assesses daily use of the light treatment device. Subjective measures of sleep are self-reported using the Pittsburgh Sleep Quality Index (PSQI) [54], Morningness Eveningness (Owl-Lark) Questionnaire (MEQ) [55], and Insomnia Severity Index (ISI) [56] at pre-treatment, mid-treatment, and post-treatment.

### Safety measures

At each weekly visit after the start of treatment (i.e., visits 4–7), participants complete a self-reported measure of physical and emotional symptoms they have experienced in the past week using the Systematic Assessment for Treatment Emergent Events (SAFTEE) as used in a previous light treatment study [57]. An unblinded research staff assesses severity of symptoms and its relevance to the light treatment to ensure no significant negative effects are associated with the participant’s light treatment. If severity meets a pre-determined threshold, the unblinded research staff alerts a PI and/or licensed clinical psychologist or a physician based on symptom nature to further assess the participant’s safety and risk for an adverse event. At each session, suicidality is also assessed using the Columbia Suicide Severity Rating Scale (C-SSRS) [42].

### Data analysis

Primary analytic cohorts will be intent-to-treat and per-protocol. To establish a relationship between the duration of daily morning light treatment (dose) and reduction in amygdala reactivity (response) from pre-treatment to mid-treatment and/or post-treatment, data will be analyzed using responses at all three times (pre-treatment, mid-treatment and post-treatment) as dependent variables using a mixed-effects longitudinal data model. Several contrasts will be explored on the EFAT (e.g., negative faces v. shapes, fear faces v. happy faces) and ERT (e.g., reappraise v. look, maintain v. look). Participants will be included as random intercepts to account for between-participant variability and time will be a predictor. First, we will graphically explore the relationship between responses and dose by time and specify how dose and time will be parameterized. For example, if the rate of change over time in response is linear and depends on dose (e.g., greater drop in amygdala reactivity over time with increasing duration of daily light pulse), we will assess it by including the interaction of time by dose (in minutes) and the main effects of time and of dose. The interaction term will allow testing if dose-dependent relationship linearly increases or decreases in time. If a nonlinear dose-dependent relationship is shown in graphs (e.g., about equal size drop in amygdala reactivity with 30 and 60 mins of daily light pulse, although greater than 15 mins of daily light pulse), it will be explored using indicators of dose groups. Time can also be included as categorical indicators to see, for example, if four weeks, relative to two weeks achieve even greater (or no greater) response than two weeks relative to pre-treatment. A flexible model including categorical time indicators and dose indicators and their interactions will allow testing for a more complicated relationship between the daily dose and reduction in amygdala reactivity from pre-treatment to mid-treatment and/or post-treatment. If the interactions are not significant based on the likelihood ratio test, we will fit the model without the interaction terms and assess for overall 30 min and 60 min dose effects vs. 15 min dose, and the overall mid- and post-treatment effects vs. pre-treatment. Regarding missing data, if “non-ignorable” patterns are detected, then “pattern mixture”, “selection models”, or other appropriate strategies will be used to examine the impact of the missing data pattern on the key outcomes [58, 59]. All analyses will include tests for sex differences, to examine sex as a biological variable.

In addition to the effect size comparison for the region of interest analysis, we will also conduct exploratory whole-brain voxel-wise analyses, employing a similar statistical approach across the entire brain in an exploratory search to generate hypotheses for subsequent study. Statistical maps for the whole brain analysis will be created using a threshold of p<0.001 with a cluster threshold of at least 10 voxels. Brain images will be entered into second-level analyses implemented in SPM12 to mirror analyses performed with extracted values. We will also conduct exploratory amygdala analyses to evaluate: 1) different sub-regions within the amygdala; 2) activation outside of amygdala using whole-brain voxel-wise analyses; and 3) using amygdala circuit seeds for functional (psychophysiological interaction analyses), resting state and effective (dynamic causal modeling) connectivity analyses.

Changes in traumatic stress symptoms and sleep will also be assessed over the course of the intervention. Specifically, we will examine the clinician measures (PSSI-5, HAM-D, HAM-A) and self-report measures (DASS-21, PCL-5, PSQI, ISI, Owl-Lark) of traumatic stress symptoms and sleep with a mixed-effects longitudinal data model as described above. Actigraphy estimates of sleep onset time (clock time of the first epoch scored as sleep in each rest interval), wake time (clock time of the last epoch scored as sleep in each rest interval), total sleep time (number of minutes scored as sleep in each rest interval), and wake after sleep onset (number of minutes scored as wake between sleep onset and wake time) will be extracted for each day, then averaged for the pre-treatment, mid-treatment, and post-treatment weeks, and analyzed similarly to clinical and self-report measures.

## Discussion

This protocol manuscript describes a single-blinded three-arm randomized controlled mechanistic trial aiming to establish a significant dose response relationship between the duration of morning light treatment and reduction in amygdala reactivity. Although front-line treatments exist for trauma-related psychopathology, additional treatment options are needed. With existing approaches, many individuals fail to receive evidence-based psychotherapies and pharmacotherapies [5, 6] and very few individuals receive a minimally adequate dose of treatment [10, 60]. Uptake of these treatments is poor for several reasons including stigma, avoidance, unpleasant side effects, and poor accessibility [11]. As a self-administered, commercially available, low risk treatment, morning light treatment has the potential to overcome these critical barriers to care and be easily scaled to meet treatment needs. Moreover, even among those who do receive psychotherapy or pharmacotherapy, a substantial minority of individuals remain symptomatic, suggesting that alternative treatments are needed [7, 8]. Morning light therapy has been found to be highly effective in treating seasonal and nonseasonal depression [61, 62] and some studies have indicated therapeutic benefit for anxiety symptoms as well [63–65]. Light therapy has also led to improvement in PTSD symptoms in two randomized trials [14, 15]. Delineating some of the mechanisms underlying the effects of morning light treatment on traumatic stress symptoms will better inform our understanding of its therapeutic potential.

This study will have limitations. First, the exclusion of individuals with current substance use disorder and with certain sleep disorders will limit the generalizability of the findings. Second, follow-up visits will not be conducted; therefore, the durability of treatment effects will not be captured. Lastly, with a pre-post fMRI design, there are issues of stability for test-retest activation in brain regions and for fMRI signal change due to scanner-related effects or measurement errors. Test-retest stability of BOLD signal is evident in studies scanning the amygdala (8 weeks– 1 year) [24, 66, 67]. To ameliorate these issues, we will measure signal to noise ratio (SNR) values extracted from ROIs for each of the fMRI sessions to confirm sufficient and similar SNR effects across all sessions. Additionally, test-retest reliability with intra-class correlations from ROIs for all participants across the sessions will also be examined.

### Conclusions and innovations

The current transdiagnostic study is the first to evaluate the underlying brain mechanisms of a wearable morning light treatment for individuals with traumatic stress. This study has the potential to uncover novel mechanistic knowledge of how morning light therapy works to identify promising treatment targets for those with traumatic stress. Furthermore, by taking an experimental therapeutics approach, this study aims to demonstrate target engagement and establish the optimal treatment dose prior to conducting a time-intensive and expensive randomized control trial. In doing so, this study will provide future researchers an understanding of what mechanisms should be targeted, allowing for refinement of current intervention approaches to enhance treatment efficacy and efficiency.

## Supporting information

S1 File (DOCX)

S1 Checklist (DOC)
