# Supplementary material for: Morning light treatment for traumatic stress: The role of amygdala reactivity study protocol
Source: PLoS One. 2022 Jun 8;17(6):e0269502. doi: 10.1371/journal.pone.0269502 (PMC9176814; doi:10.1371/journal.pone.0269502)
Supplement: S1 File — (DOCX) [file pone.0269502.s001.docx]

**Morning Light Treatment for Traumatic Stress:**

**The Role of Amygdala Reactivity**

Protocol Number: HUM00161267

National Clinical Trials (NCT) Number: NCT04117347

Principal Investigator: Helen Burgess, PhD

Co-Principal Investigator: Alyson Zalta, PhD

Initial Version: 09 April 2019

Current Version: v12.0

March 3, 2022

**Statement of Compliance**

This trial will be carried out in accordance with the United States (US) Code of Federal Regulations (CFR) applicable to clinical studies (45 CFR Part 46, 21 CFR Part 50, 21 CFR Part 56, 21 CFR Part 312, and/or 21 CFR Part 812) and research best practices. The PIs and all study team members who are responsible for the conduct, management, or oversight of NIH-funded clinical trials will complete Human Subjects Protection and best practices training.

The protocol, informed consent document, recruitment materials, and all participant materials will be submitted to the IRB for review and approval. Approval of both the protocol and the consent documents will be obtained before any participant is consented. Any amendment to the protocol will be submitted for review and approval by IRBMED before the changes are implemented to the study. All changes to the consent form(s) will be IRB approved; a determination will be made regarding whether a new consent needs to be obtained from participants who provided consent, using a previously approved consent form.

Amendments

| **Date** | **Version** | **Section(s)** | **Changes** |
| --- | --- | --- | --- |
| Oct. 8, 2019 | 2.0 | 1.3 | Updated schedule of activities to clarify timing of self-report surveys and safety assessments |
|  |  | 3.1.3.3 | Added info on Emotional Regulation Task for fMRI |
|  |  | 5.1 | Changed overall design from “double” to “single” blinded to correct a mistake |
|  |  | 6.1 | Changed trauma criterion to remove within 5-year time frame |
|  |  | 6.2 | Clarified “significant disease” criterion to mention uncontrolled CVD; added beta blockers and opioids and SNRIs to contraindicated meds; expanded “acute suicidal ideation” criterion to include suicidal behaviors within last 6 months |
|  |  | 6.3 | Clarified alcohol and drug use as they relate to withdrawals |
|  |  | 6.4 | Indicated that community-based recruitment would include Ann Arbor/Ypsi and greater Detroit regions |
|  |  | 6.4.5 | Added section on Pre-screening via REDCap & telephone surveys, and the emailing of materials to prospective participants |
|  |  | 6.5 | Clarified that travel stipends are only available for visits to the research clinic/Rachel Upjohn building |
|  |  | 7.5/7.6 | Added beta blockers and opioids and SNRIs to the list of contraindicated meds |
|  |  | 9.5/9.6 | Added Beck Scale for Suicidal Ideation |
|  |  |  | Added Demographics form, Work & Social Adjustment Form, fMRI screening form, and Treatment Expectation and Satisfaction forms; Insomnia Severity Index; Morningness/Eveningness Questionnaire |
|  |  | 9.71 | Added section on fMRI practice |
|  |  | 9.7.4 | Added section on Emotional Regulation Task |
|  |  | 9.9.1 | Clarified screening process for fMRI as relates to pregnancy and drug screening |
|  |  | 12.2.2/12.3.3.1 | Indicated that video files will be stored on MBox |
|  |  | 12.3.2 | Added section naming the Independent Safety Monitor |
|  |  | 12.4 | Added ERT and WSAS to abbreviations list |
|  |  | 13 | Added references for BSSI and WSAS questionnaire |
| Jan. 13, 2020 | 3.0 | 1.3; 5.1 | Added footnote to SOA and language to section 5.1 that allows for Visit 2 activities to occur on two separate days. |
|  |  | 6.5 | Updated incentive schedule to reflect possibility of receiving an additional travel stipend related to visit 2 schedule |
| Feb. 4, 2020 | 4.0 | 6.2 | Updated Exclusion Criteria to specify excluding for *significant* light treatment use and/or use in the past year |
| Feb. 25, 2020 | 5.0 | 6.4.3; 6.4.4 | Updated recruitment strategies to specify flyering at the VA and removed flyering at the VA as a future recruitment strategy. |
| March 5, 2020 | 6.0 | 1.3 | Updated SOA to indicate that the PCL-5 and DASS will also be collected at lab visits 4 and 6; updated Table of Contents |
| March 30, 2020 | 7.0 | 3.1.5.1 | Removed language that indicated only the clinician would be filmed. |
|  |  | 6.5 | Outlined additional travel stipend amount depending on distance which participant will be traveling to the lab. |
| September 10, 2020 | 8.0 | 6.4.3 | Updated to reflect that IRB approved study fliers will be posted on Wayne State University campus. |
| October 13, 2020 | 9.0 | 1.3, 9.9.1 | Added drug test to lab visit 2 |
| July 1, 2021 | 10.0 | 6.4.4 | Added Data Direct/EMERSE as recruitment tools |
|  |  | 12.2.1.1 | Specified data retention plan for data obtained from the EMR (used for recruitment) |
|  |  | 12.4 | Updated Abbreviations List with PHI |
| July 16, 2021 | 11.0 | 6.2 | Updated and modified Exclusion Criteria to specify excluding for significant light treatment use and/or use in the **past 6 months** |
| March 3, 2022 | 12.0 | 6.4.7 | Added option of free cabs rides for participants with no access to timely public transportation or a car/ride with family or friends. |

Table of Contents

[1 Protocol Summary 1](#_Toc76031293)

[1.1 Synopsis 1](#_Toc76031294)

[1.2 Schema 2](#_Toc76031295)

[1.3 Schedule of Activities 3](#_Toc76031296)

[2 Introduction 5](#_Toc76031297)

[2.1 Study Rationale & Background 5](#_Toc76031298)

[2.2 Innovation 6](#_Toc76031299)

[3 Risk/Benefit Assessment 6](#_Toc76031300)

[3.1 Known Potential Risks 7](#_Toc76031301)

[3.2 Known Potential Benefits 9](#_Toc76031302)

[3.3 Assessment of Potential Risks & Benefits 9](#_Toc76031303)

[4 Objectives & Endpoints 10](#_Toc76031304)

[5 Study Design 10](#_Toc76031305)

[5.1 Overall Design 10](#_Toc76031306)

[6 Study Population 11](#_Toc76031307)

[6.1 Inclusion Criteria 11](#_Toc76031308)

[6.2 Exclusion Criteria 11](#_Toc76031309)

[6.3 Lifestyle Considerations 12](#_Toc76031310)

[6.4 Strategies for Recruitment & Retention 12](#_Toc76031311)

[6.5 Participant Incentives 14](#_Toc76031312)

[6.6 Screen Failures 15](#_Toc76031313)

[7 Study Intervention 15](#_Toc76031314)

[7.1 Description 15](#_Toc76031315)

[7.2 Administration & Dosing 15](#_Toc76031316)

[7.3 Fidelity 15](#_Toc76031317)

[7.4 Measures to Minimize Bias 16](#_Toc76031318)

[7.5 Concomitant Therapy 16](#_Toc76031319)

[7.6 Contraindicated Therapy 16](#_Toc76031320)

[8 End-of-Intervention/End-of-Study 16](#_Toc76031321)

[8.1 Discontinuation of Intervention 16](#_Toc76031322)

[8.2 Participant Discontinuation or Withdrawal from Study 17](#_Toc76031323)

[9 Study Assessments & Procedures 17](#_Toc76031324)

[9.1 Eligibility Confirmation 17](#_Toc76031325)

[9.2 Vision Testing 17](#_Toc76031326)

[9.3 Psychological Assessment 17](#_Toc76031327)

[9.4 Sleep Assessment 18](#_Toc76031328)

[9.5 Clinician-administered* & Self-reported Assessments 18](#_Toc76031329)

[9.6 Other Self-reported Assessments 18](#_Toc76031330)

[9.7 fMRI 18](#_Toc76031331)

[9.8 Treatment expectation & Treatment Satisfaction 19](#_Toc76031332)

[9.9 Other Research Procedures 19](#_Toc76031333)

[9.10 Safety Assessments 20](#_Toc76031334)

[10 Adverse Events 21](#_Toc76031335)

[10.1 Definitions 21](#_Toc76031336)

[10.2 Classification 21](#_Toc76031337)

[10.3 Reporting 22](#_Toc76031338)

[10.4 Unanticipated Problems 22](#_Toc76031339)

[11 Statistical Considerations 23](#_Toc76031340)

[11.1 General Approach 23](#_Toc76031341)

[11.2 Analysis of Primary Outcome 23](#_Toc76031342)

[11.3 Exploratory Analyses 24](#_Toc76031343)

[12 Supporting Documentation & Oversight Considerations 24](#_Toc76031344)

[12.1 Regulatory, Ethical & Study Oversight 24](#_Toc76031345)

[12.2 Data Retention 25](#_Toc76031346)

[12.3 Safety Oversight 26](#_Toc76031347)

[12.4 Abbreviations 27](#_Toc76031348)

[13 References 28](#_Toc76031349)

# Protocol Summary

## Synopsis

| **Title:** | Morning Light Treatment for Traumatic Stress: The Role of Amygdala Reactivity |
| --- | --- |
| **Study Description:** | Participants will be enrolled in a 3-arm randomized controlled trial of a 4-week self-administered wearable morning light treatment to evaluate the dose-response association between amount of light therapy (15, 30 or 60 minutes per day) and the degree of brain reactivity in the amygdala in response to an emotional faces fMRI task. |
| **Primary Objective** | Average blood-oxygen-level-dependent (BOLD) percent signal change in the bilateral amygdala from baseline to week 2 and baseline to week 4 of treatment. |
| **Study Population:** | Individuals with traumatic stress and significant distressing symptoms |
| **Phase:** | n/a |
| **Description of Sites/Facilities Enrolling Participants:** | University of Michigan Sleep and Circadian Research Lab & North Campus fMRI suite |
| **Description of Study Intervention:** | Daily self-administered morning light therapy delivered using a commercially available device, the Re-Timer. |
| **Study Duration:** | 3 years |
| **Participant Duration:** | 5 weeks |

## Schema

The figure below shows a sample protocol for an individual whose average sleep schedule is 12-8am:


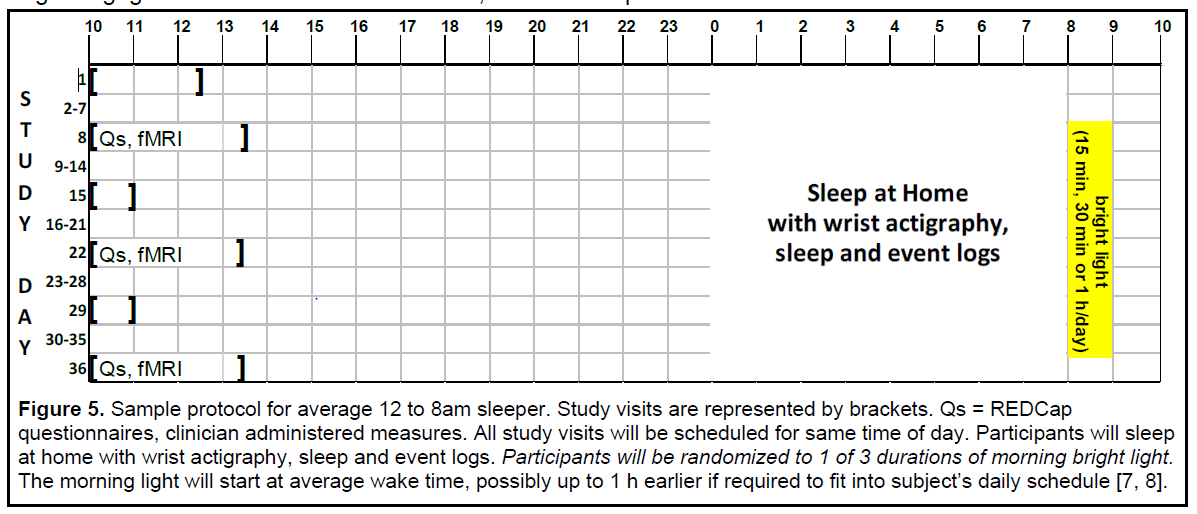


## Schedule of Activities

| **Study Phase** | **Screening & Baseline ^5^** | | | | | | | | | **Intervention** | | | | | | | | | | | | | | | | | | | | | | | | | | | |
| --- | --- | --- | --- | --- | --- | --- | --- | --- | --- | --- | --- | --- | --- | --- | --- | --- | --- | --- | --- | --- | --- | --- | --- | --- | --- | --- | --- | --- | --- | --- | --- | --- | --- | --- | --- | --- | --- |
| **Day Number ^1^** | **0** | **1** | **2** | **3** | **4** | **5** | **6** | **7** | **8** | **9** | **10** | **11** | **12** | **13** | **14** | **15** | **16** | **17** | **18** | **19** | **20** | **21** | **22** | **23** | **24** | **25** | **26** | **27** | **28** | **29** | **30** | **31** | **32** | **33** | **34** | **35** | **36** |
| **Visit Number** | **v1** | **v2^6^** |  |  |  |  |  |  | **v3** |  |  |  |  |  |  | **v4** |  |  |  |  |  |  | **v5** |  |  |  |  |  |  | **v6** |  |  |  |  |  |  | **v7** |
| Informed consent | X |  |  |  |  |  |  |  |  |  |  |  |  |  |  |  |  |  |  |  |  |  |  |  |  |  |  |  |  |  |  |  |  |  |  |  |  |
| Eligibility Confirmation | X | x |  |  |  |  |  |  |  |  |  |  |  |  |  |  |  |  |  |  |  |  |  |  |  |  |  |  |  |  |  |  |  |  |  |  |  |
| Vision Testing | X |  |  |  |  |  |  |  |  |  |  |  |  |  |  |  |  |  |  |  |  |  |  |  |  |  |  |  |  |  |  |  |  |  |  |  |  |
| Psychological assessment |  | X |  |  |  |  |  |  |  |  |  |  |  |  |  |  |  |  |  |  |  |  |  |  |  |  |  |  |  |  |  |  |  |  |  |  |  |
| Accelerometry |  | X | X | X | X | X | X | X | X | X | X | X | X | X | X | X | X | X | X | X | X | X | X | X | X | X | X | X | X | X | X | X | X | X | X | X | X |
| PSQI |  |  |  |  |  |  |  |  | X |  |  |  |  |  |  |  |  |  |  |  |  |  | X |  |  |  |  |  |  |  |  |  |  |  |  |  | X |
| Daily log ^2^ |  | X | X | X | X | X | X | X | X | X | X | X | X | X | X | X | X | X | X | X | X | X | X | X | X | X | X | X | X | X | X | X | X | X | X | X | X |
| Urine screen ^3^ | X | x |  |  |  |  |  |  | X |  |  |  |  |  |  |  |  |  |  |  |  |  | X |  |  |  |  |  |  |  |  |  |  |  |  |  | X |
| Breathalyzer | X | X |  |  |  |  |  |  | X |  |  |  |  |  |  | X |  |  |  |  |  |  | X |  |  |  |  |  |  | X |  |  |  |  |  |  | X |
| Clinician-administered assessments |  |  |  |  |  |  |  |  | X |  |  |  |  |  |  |  |  |  |  |  |  |  | X |  |  |  |  |  |  |  |  |  |  |  |  |  | X |
| Self-report questionnaires |  |  |  |  |  |  |  |  | X |  |  |  |  |  |  | X^7^ |  |  |  |  |  |  | X |  |  |  |  |  |  | X^7^ |  |  |  |  |  |  | X |
| fMRI ^4^ |  |  |  |  |  |  |  |  | X |  |  |  |  |  |  |  |  |  |  |  |  |  | X |  |  |  |  |  |  |  |  |  |  |  |  |  | X |
| Randomization |  |  |  |  |  |  |  |  | X |  |  |  |  |  |  |  |  |  |  |  |  |  |  |  |  |  |  |  |  |  |  |  |  |  |  |  |  |
| Treatment expectation |  |  |  |  |  |  |  |  | X |  |  |  |  |  |  |  |  |  |  |  |  |  |  |  |  |  |  |  |  |  |  |  |  |  |  |  |  |
| Light therapy |  |  |  |  |  |  |  |  |  | X | X | X | X | X | X | X | X | X | X | X | X | X | X | X | X | X | X | X | X | X | X | X | X | X | X | X | X |
| Treatment fidelity – daily contact |  |  |  |  |  |  |  |  |  | X | X | X | X | X | X | X | X | X | X | X | X | X | X | X | X | X | X | X | X | X | X | X | X | X | X | X | X |
| Treatment fidelity – data download & feedback |  |  |  |  |  |  |  |  |  |  |  |  |  |  |  | X |  |  |  |  |  |  | X |  |  |  |  |  |  | X |  |  |  |  |  |  | X |
| AE Assessment (SAFTEE/CSSRS) | x | x |  |  |  |  |  |  | X |  |  |  |  |  |  | X |  |  |  |  |  |  | X |  |  |  |  |  |  | X |  |  |  |  |  |  | X |
| Treatment satisfaction |  |  |  |  |  |  |  |  |  |  |  |  |  |  |  |  |  |  |  |  |  |  |  |  |  |  |  |  |  |  |  |  |  |  |  |  | X |
| *1 – Exact day number can vary by up to one week; 2 – Daily log will capture information about sleep time, treatment time, medication use, etc.; 3 – Urine drug screen administered via dipstick by study team at baseline and urine pregnancy screen as part of standard fMRI lab standard operating procedures at v3, v5 and v7; 4 – fMRI must occur within 7 days of clinician-administered assessments; 5 – Final screening and eligibility confirmation occur on day 0/v1 and the start of the week-long baseline period begins at v2; 6 – Visit 2 activities may be split across 2 days separated by no more than 1 month; 7 – Only the PCL-5 and DASS will be administered at these visits* | | | | | | | | | | | | | | | | | | | | | | | | | | | | | | | | | | | | | |

# Introduction

## Study Rationale & Background

### Traumatic Stress and the Need for Additional Treatment Strategies That Target Brain Mechanisms

The mental health burden of trauma is costly for individuals and society [37]. Evidence from a national sample of U.S. adults using the DSM-5 criteria, showed that 89.7% of individuals had been exposed to a traumatic event, including 53.1% of individuals exposed to direct interpersonal violence victimization [38]. Trauma leads to a wide variety of mental health problems including posttraumatic stress disorder (PTSD), depression, anxiety, substance use, and suicidality [23, 24]. Although front-line psychotherapies and pharmacotherapies exist for traumatic stress, evidence suggests that many individuals fail to receive treatment, let alone a therapeutic dose [25-28]. Uptake of these treatments is poor for several reasons including stigma, avoidance, unpleasant side effects, and poor accessibility [29]. Moreover, a substantial proportion of affected individuals remain symptomatic despite treatment [30-32]. New treatments are needed for traumatic stress that can overcome these critical barriers to care while targeting the underlying biological mechanisms of the pathology.

One of the central brain regions implicated in traumatic stress is the amygdala [39, 40]. The amygdala plays a critical role in emotional memory and the acquisition of fear responses [41], which are intimately related to the clinical manifestation of traumatic stress symptoms. Research has shown increased amygdala function during exposure to traumatic reminders [42, 43], and amygdala hyperactivity to salient cues has been linked to traumatic stress symptoms including posttraumatic stress symptoms [11, 12], depressive symptoms [13], and anxiety symptoms [14]. In a rodent model of PTSD, deep brain stimulation of the amygdala (inhibits amygdala function) reduced PTSD-like behaviors, demonstrating a causal link between changes in amygdala function and PTSD-like behavior [19, 20]. There is currently an ongoing trial of deep brain stimulation of the amygdala in humans (NCT02091843) and a published case report from the first patient (treatment resistant to psychotherapy and pharmacotherapy) showed substantial clinical improvements [21]. These studies suggest that changes in amygdala function are causally linked to changes in PTSD-like / PTSD symptoms. Moreover, several studies have demonstrated decreased amygdala activation as a result of successful treatment of PTSD [15, 16], depression [41], and anxiety disorders [44]. Thus, decreasing amygdala reactivity appears to be a critical treatment target for traumatic stress.

### The Potential of Morning Bright Light as A Novel Treatment for Traumatic Stress

Morning bright light treatment has good potential as a novel, non-invasive, low risk treatment for trauma-related disturbances in mood and arousal. With depression, the most recent meta-analyses reveal that morning bright light yields medium treatment effect sizes in seasonal depression (d=0.54, [17]) and nonseasonal depression (d=0.62, [18]). Antidepressant effects became statistically significant as early as 2 weeks after the initiation of a daily morning bright light treatment [17, 18]. Few studies have examined morning bright light treatment for anxiety, but there are some indications of anxiolytic effects [34-36]. In terms of post-traumatic stress disorder (PTSD), a recently completed morning bright light treatment trial has reported positive effects versus placebo (clinical trial NCT00701064). We have also shown in a placebo-controlled pilot trial (n=13) that a 4-week wearable morning bright light treatment (Re-Timer) for people with probable PTSD led to clinical improvements in PTSD symptoms. While bright light is associated with some side effects (headache, eyestrain, nausea, agitation [45]), these often spontaneously remit [45, 46], and patients rarely discontinue due to side effects [46]. Bright light devices (with UV filter) are considered safe with no changes in ophthalmologic exams observed after 6 years of daily use (in fall and winter months) [47].

### Light Reaches the Amygdala via Direct and Indirect Pathways, Can Reduce Amygdala Reactivity

The primary circadian photoreceptors in the eye are specialized cells known as intrinsically photosensitive retinal ganglion cells (ipRGCs) [48]. IpRGCs express melanopsin, a photopigment that can respond to light directly [49]. IpRGCs also receive inputs from the rods and cones through conventional retinal circuitries [50]. The combination of intrinsic (melanopsin) and extrinsic (rod and cone) inputs enable ipRGCs to signal a large dynamic range of light levels in the environment (by a factor of 10 billion; from dim starlight to bright sunlight) [51]. IpRGCs transmit the light signal to various brain targets, including the amygdala [2]. The light signal reaches the amygdala directly via the ipRGCs [2], and also indirectly via other brain targets such as the suprachiasmatic nuclei, which is the central circadian clock in the brain [2, 33]. During fMRI scans with cognitive tasks, the application of a light signal immediately led to greater amygdala activation [9]. This effect was considered independent from the ongoing cognitive brain activity [9]. There is only one study that has examined amygdala activity before and after a bright light treatment. In this study, 30 healthy male participants received a 3-week morning bright white light treatment (30 min/day) of varying intensity, from 100-11,000 lux. The brighter the white light, the greater the pre- to post-treatment reduction in amygdala reactivity observed during an emotional faces fMRI task [10]. Thus, there is compelling evidence to suggest morning bright light treatment may reduce traumatic stress by inducing a reduction in amygdala reactivity. Although the amygdala is just one part of a larger neural network involved in the pathophysiology of traumatic stress, current evidence supports the hypothesis that light treatment can reduce traumatic stress symptoms via direct effects on amygdala function. If so, potential synergies with treatments targeting other nodes in this network may amplify the impact of this work and the importance of this potentially novel, non-invasive and scalable treatment.

## Innovation

This project will be the first to establish if the anatomical links between ipRGCs and the amygdala translate into clinically meaningful changes in amygdala reactivity using morning light treatment. Amygdala hyperactivity has been associated with many different types of pathology that result from trauma exposure (i.e., PTSD, depression, anxiety) [23]; thus, we expect that if morning light treatment has a meaningful therapeutic effect on amygdala reactivity, it will have a broad impact on traumatic stress symptoms, potentially leading to a shift in clinical practice. The use of a wearable light device, the Re-Timer, which is user-friendly and enables the optimization of the therapeutic light wavelength, adds to the innovation by facilitating successful translation into future studies and practice.

# Risk/Benefit Assessment

Overall, this study poses no more than minimal risk to the participant. The known risks fall into the following categories: risks associated with the research assessments, risk associated with the intervention, risks associated with worsening mental or emotional state, and risk associated with breaches of privacy and confidentiality.

## Known Potential Risks

### Clinical assessments

The diagnostic and clinical interviews (SCID, HAM-D, etc.) and questionnaires are time consuming, and probe potentially sensitive topics like trauma history and suicidal ideation, etc. Some individuals might feel temporary distress at having to answer questions about their personal history. The instruments used in this study are well-tested and not known to cause persistent problems or distress; however, individuals are instructed that they can refuse to answer any question with which they are uncomfortable.

### Worsening mental or emotional state

Some participants may show a worsening of psychological symptoms or suicidality during the course of the study period, regardless of assigned treatment arm. Among traumatized individuals, it is common for symptoms of emotional distress, including depression, hopelessness, and suicidal ideation to wax and wane. Thus, this risk is inherent in the population. We do not believe these risks are increased as a function of being enrolled in this study; if anything, we expect these symptoms to decrease as a function of being in the study. Participants will complete measures of anxiety, depression and suicidal ideation throughout the study, and be monitored by study staff and investigators for clinically significant changes.

### fMRI

The potential risks such of undergoing a functional MRI are typically rare, and generally not considered dangerous or life threatening. They include:

- Discomfort or anxiety from being in the confined space of the MRI scanner
- The experience of peripheral nerve stimulation secondary (PNS) to fast imaging sequences, such as those employed in this study. PNS is often described as a light touching sensation on the skin surface and may cause mild discomfort, but is not harmful to the participant
- Hearing damage due to loud noises produced by the scanner
- Discovery of a previously unknown minor or significant lesion in the brain (e.g. a tumor) that might require additional follow-up
- Injury from objects accelerated by the strong magnetic field of the magnet, striking the participant; or metallic substances on the skin or foreign bodies implanted deliberately or accidentally in the participant that acquire kinetic or thermal energy from the magnetic or radiofrequency emissions of the MRI, causing tissue injury to the participant
- Temporary, slight dizziness or light-headedness when they come out of the scanner

The University of Michigan’s fMRI Lab has well-established protocols for assessing and reducing risk, and addressing any arising complications. Participants in this study are screened multiple times for contraindications to MRI by the study staff and the fMRI Lab technicians. Women of childbearing potential are asked to complete a urine pregnancy test as part of standard fMRI Lab operating procedures. Once in the scanner, participants are provided with custom pads and pillows and ear plugs to make the scanning process more comfortable. The machine is operated in accordance with FDA guidelines so the risk of PNS is low. Participants can communicate with study and fMRI lab staff via an intercom, and they can stop the testing at any time.

#### fMRI and pregnancy

According to the NIMH Council Workgroup on MRI Research and Practices (September, 2005), “there is no known risk of MR brain scanning of a pregnant woman to the developing fetus for scanning at 4T or less, and no known mechanism of potential risks under normal operating procedures.” Nevertheless, participants should be warned about potential risks not yet discovered.

#### fMRI and incidental findings

During the formal consent process, all participants will be informed about the potential risks of discovering an incidental finding or abnormality on their MRI scan. If an abnormality is found in a participant’s MRI scan, Dr. Duval (Co-I) will contact the participant and refer the participant for medical follow-up for the problem if the participant requests, including a referral to a primary care physician. If the participant has a primary care physician, Dr. Duval will contact the participant’s doctor, at the request and with permission from the participant, to inform him/her of the finding on the MRI scan and to help him/her get the participant appropriate follow-up. The decision as to whether to proceed with further examination and/or treatment lies solely with the participant and his/her primary care physician.

#### Emotional Face Assessment Task (EFAT) & Emotional Regulation Task (ERT)

There is little risk associated with completing either the EFAT or ERT. Though unlikely, some individuals might report mild subjective anxiety. Co-investigator, Dr. Duval (licensed clinical psychologist) will be available during the task and MRI in the event of a more severe response such as anxiety/panic attack or distressing levels of anxiety. Participants will have an opportunity to preview examples of the images prior to getting into the scanner. If the study team has any doubts about relief of anxiety/distress, Dr. Duval will conduct follow-up telephone calls 1-3 days after the fMRI session to confirm the transient nature of the participant’s reaction.

### Morning Light Treatment

Reported side effects of morning light treatment are typically mild and short-lived [45, 46], and are rarely cause for discontinuation of treatment. Most commonly reported are headache, eyestrain, nausea and agitation. [45]

Some participants might experience jet lag type symptoms (e.g. transient sleepiness, fatigue and headache) like those seen when crossing 1 or 2 time zones because of circadian phase shifts secondary to morning light treatment. These symptoms are short-lived and are akin to what one might feel on a Monday morning after a late weekend.

A rare but serious side effect is mania in bipolar patients who overexpose themselves to the light [45, 46], but bipolar depressives are excluded from this study and all participants will be provided with strict instructions to limit their light treatment to 15 minutes, 30 minutes or 1 hour/day. Note that mania in response to morning light treatment is so rare that light treatment has even been clinically tested in bipolar patients and found to be safe [96].

The light treatment device used in this study, the Re-Timer is commercially-available, and meets all international ultraviolet and blue light hazard safety standards. The emitted light, while brighter than most indoor light, is much dimmer than sunlight on a bright day; and the device has an auto-off feature after 1 hour of use.

### Break of privacy or confidentiality

The collection of study data poses a potential risk of loss of confidentiality around sensitive information such as psychiatric status, history of substance abuse, etc. Participants will be informed in the consent document that confidentiality will be limited in cases where the participant reveals intentions to harm themselves or others, and the investigator feels that the proper authorities may need to be notified in order to prevent the occurrence of harm to the participant, or others. Interviews will be conducted by experienced mental health workers who will maintain confidentiality, and all data from interviews and questionnaires will be coded so as to conceal the identity of the participant.

Likewise, research data will be labeled with study codes rather than personal identifiers (e.g name, date of birth, MRN, etc.). Identifiers and PHI are maintained in a HIPAA-compliant database, and documents are stored in a locked file cabinet. Access to both electronic and physical data is restricted based on job responsibility.

#### Video Recording

The clinician-administered assessments will be videotaped. The recording equipment will be trained on both the clinician and the participant, so participants may be identified by their face and voice. Recordings are considered part of the research record and will be afforded the same level confidentiality. The recordings are not transcribed, but rather reviewed for training purposes and to assess clinician compliance with the administration guidelines. The video files will be stored in the study database on Michigan Medicine servers and will be available only to authorized study staff.

### Unknown Risks

As with any research study, there might be risks that are unknown to the study team. The research and safety assessments, including both systematic and ad hoc monitoring, should contribute to capture of most adverse events and allow for the documentation of any heretofore unknown risks.

## Known Potential Benefits

Participants may experience an improvement in their traumatic stress symptoms during the study either through the act of sharing information about their trauma or through the treatment itself, or even by their mere involvement with the study.

The potential benefit to others is that the study may advance our understanding of how morning light therapy may lead to therapeutic benefit for individuals affected by traumatic stress.

## Assessment of Potential Risks & Benefits

Given that the potential risks associated with participating are minor and the potential benefits are considerable, it is believed that the risks are greatly outweighed by the benefits.

# Objectives & Endpoints

This study is a part of a larger proposal under the NIH R61/R33 mechanism which requires specific Go-No Go Criteria to transition between the two phases of the proposal. This protocol is specific to the R61 Phase. The specific aim and **objective for this phase** (i.e. study) is to **establish a significant dose response relationship between duration of morning light pulse and reduction in amygdala reactivity** from baseline to week 2 and/or week 4.

The **endpoint is whole-brain oxygen-level-dependent (BOLD) fMRI signal** using a priori regions of interest **specific to the bilateral amygdala**.

# Study Design

## Overall Design

- Single-blinded randomized controlled mechanistic trial
- Parallel groups
- 3 study arms based on light treatment duration: 15-minutes, 30-minutes or 60-minutes/day
- 4-weeks of morning bright light treatment delivered via Re-Timer device

This is a 5-week study (1-week of baseline and 4-weeks of intervention) that includes a screening visit plus 6 additional study visits to evaluate outcomes and monitor progress. Potential participants are pre-screened for major exclusion criteria using a combination of online and telephone surveys. On Day 0 (Visit 1) individuals are consented and eligibility confirmed using several self-report questionnaires. Final eligibility is confirmed after the clinician interviews on visit 2 do not reveal any exclusionary psychiatric conditions (see below).

Participants return to the lab for study visit 2 for a battery of psychological assessments to characterize the sample, and to begin the week-long baseline period (see Section 1.3 - Schedule of Activities). At this visit, participants are provided with their study materials (Actiwatch Spectrum, daily log, instructions to sleep according to a normal schedule, etc.). To minimize burden participant burden and to accommodate calendar challenges (e.g. time change), the activities for this visit may be split across 2 days such that the psychological assessments are done on one day and the distribution and training of study materials occur on a second day within 1 months’ time.

On Day 8 (Visit 3) participants return to the lab for randomization and baseline outcome assessments. Morning light treatment begins on the morning of Day 9. Thereafter, participants come to the research lab weekly to assess adherence to the study procedures and intervention, and monitor for side effects. Outcome assessments occur on Day 8, Day 22 (week 2 of intervention) and Day 36 (week 4 of intervention). The exact day number of study visits and activities can vary by up to a week; and we will try to schedule study visits for the same time of day. While the plan is to schedule the fMRI for the same day as the psychological assessments, we do allow for a 7-day window in cases where same-day scheduling is not possible.

Data collection is expected to last approximately 19 months with plans to enroll approximately 3 participants a month with possible exceptions for lower enrollment in November and March when offset and onset of daylight savings time can confound circadian rhythms and normal sleep habits.

# Study Population

We plan to enroll 66 individuals with a recent history of DSM-5 Criterion A trauma and reported co-morbid mood and arousal symptoms (as measured by the DASS questionnaire). These criteria are designed to include a wide variety of people with traumatic stress to maximize generalizability while ensuring that treatment and outcomes measures are not significantly affected by potential confounding variables. Additionally, we believe that this approach will lead to a transdiagnostic sample that shares the common feature of amygdala reactivity [11-14]. Diagnostic information will be collected via the Structured Clinician Interview for DSM Disorders (SCID-5-RV) [57] during Visit 2.

## Inclusion Criteria

- Age 18-60 years old
- Experienced a DSM-5 Criterion A trauma
- Depression Anxiety Stress Scale score (DASS) > 22
- Two or more hyperarousal symptoms
- Normal or corrected to normal vision
- Right-handed
- Fluency in English
- Physically able to travel for study visit attendance
- Stable psychotropic and other medication use for at least 30 days prior to enrollment, and throughout the study period

## Exclusion Criteria

- Significant chronic disease (e.g. uncontrolled diabetes or cardiovascular disease, advanced liver disease, cancer, etc.)
- Retinal pathology or eye disease like current cataracts or glaucoma, or history of eye surgery
- Currently taking photosensitizing medications
- Currently taking benzodiazepines, selective serotonin re-uptake inhibitors (SSRIs), SNRIs, beta blockers or opioids for their dampening effects on amygdala reactivity
- History of significant light treatment use OR history of light treatment within the past 6 months
- Lifetime psychotic or bipolar disorder
- Current other significant psychological disorder (e.g. OCD)
- Acute suicidal ideation or suicidal behaviors within the last 6 months
- Substance use disorder within last 3 months
- Diagnosed with obstructive sleep apnea, restless leg syndrome, and/or narcolepsy OR at high risk for any of these disorders
- Severe hearing problem
- Intellectual disability or serious cognitive impairment
- History of traumatic brain injury, e.g. any loss of consciousness; any loss of memory for events immediately before or after injury; any alteration in mental status at time of the incident; or focal neurological deficits that may or may not be transient
- Inability to tolerate enclosed spaces (e.g. the MRI machine)
- Ferrous-containing metals within the body
- Pregnant, trying to get pregnant, or breastfeeding
- Current or recent disruptions to normal sleep schedule: e.g. night work or travel outside the Eastern time zone within the past month
- Current psychotherapy
- Epilepsy

## Lifestyle Considerations

### Alcohol and Drug Use

Participants are asked to refrain from alcohol consumption for 24 hours prior to their study visits. Any participant breathalyzing positive may be:

- Rescheduled to start the study anew if positive test occurs at Visit 2 or 3, prior to start of light treatment
- Withdrawn from the study if positive test occurs at Visits 4-7, after light treatment has started.

Additionally, participants are asked to refrain from taking illicit drugs for the duration of their study enrollment. Any participant testing positive for drug use may be withdrawn from the study as described above.

Test results will remain confidential.

### Sleep-wake patterns

Additionally, participants are asked to maintain their usual sleep-wake patterns as documented during the initial week of activity monitoring, and to refrain from napping in the 4 hours immediately after light therapy. Participants might be asked to wake up an hour earlier than usual to accommodate the light treatment in context of their life schedule (e.g. work or childcare obligations).

### Medication and Concomitant therapies

Participants are asked to maintain their current (from time of enrollment) treatments including all medications and non-pharmacological strategies throughout their participation in the study.

### Naps

Participants are asked not nap within 4 hours of completing their treatment.

## Strategies for Recruitment & Retention

We will use a multi-pronged recruitment approach to target both community members and clinical populations with traumatic stress living in Southeastern Michigan.

### Online Recruitment

Our online recruitment strategies will include craigslist.com, Facebook, and umhealthresearch.org, the University of Michigan Research Registry. We will work with MICHR’s Recruitment Core to design targeted ads to specific geographical areas (including, but not limited to the surrounding Ann Arbor/Ypsilanti and greater Detroit regions), age groups, and multiple races and ethnicities.

### Dr. Duval

Dr. Duval has an ongoing non-treatment study for individuals with PTSD. Individuals who do not meet the diagnostic criteria for PTSD yet still report traumatic stress and those individuals who have completed the study will be offered the opportunity to participant in the current protocol and/or speak with the study team.

### Flyers

We will distribute flyers to local clinics and local support groups, including but not limited to those in the surrounding Ann Arbor/Ypsilanti and greater Detroit regions. We will also distribute flyers to the Ann Arbor VA (where Dr. Duval has a Without Compensation [WOC] appointment). We will post fliers on the Wayne State University campus.

### Data Direct/EMERSE

We will use two of the online self-serve tools provided by the University of Michigan Data Office for Clinical and Translational Research to access clinical data as a means of identifying potential participants with a history of trauma or PTSD. Once identified, these individuals will be approached initially via an introductory letter, email, or text inviting them to contact the study team for information.

### Possible future strategies

If our initial strategies fail to generate sufficient enrollment numbers to achieve our accrual goals, we will consider alternatives, with no specific plan as of yet. We will submit an amendment before embarking on any additional strategies.

### Pre-screening

Prospective participants will participate in a series of pre-screening surveys: first they complete an online survey to determine basic eligibility followed by a telephone interview during screening questions for trauma history, psychiatric disorders, and other potentially sensitive criteria are discussed. Once deemed eligible at this stage, individuals are scheduled for an in-person screening visit at which additional screening procedures will occur. In advance of this visit they are sent an example study protocol (i.e. schedule) and a pre-study sleep log.

### Retention

We use a number of approaches to promote retention of participants throughout the study:

- Schedule all study visits on Day 1
- Daily outgoing communication (e.g. text, phone call, etc.) to support intervention adherence
- Weekly visits to review Re-Timer use and daily logs with participants
- Provide monetary incentive and travel stipend per visit plus a bonus for completing the entire study.
- Offer free cab rides to participants with no access to timely public transportation or a car/rides with friends and family.

During weekly lab meetings, the team will review overall participant statuses, as well as enrollment numbers and recruitment sources, and the number of withdrawals and lost-to-follow ups to allow for adjustments in the recruitment strategy as needed.

In aggregate, these strategies have proven successful in our other studies of morning light treatment. If, despite our efforts, enrollment is lacking, we will work with Co-I Dr. Abelson who has a relationship with Wayne State University Stress, Trauma & Anxiety Research Clinic to expand our reach into the Detroit area. And, we will work with the Michigan Community Outreach Program and the NIMH Outreach Partnership Program to help disseminate educational and study information to the public as a means of promoting research participation.

## Participant Incentives

Participants are eligible for up to $1260.00 in incentive payments. The payment plan was designed to partially compensate individuals for the burden of the study visits and associated travel costs, as well as promote study completion by including a bonus for finishing the protocol. Participants will be paid as the study progresses for those parts of the study completed.

- Visit 1 - $20 for screening visit – eligibility confirmation and consent
- Visit 2 – Receive study materials, no incentive
- Visit 3 - $225
  - $75 for completing the baseline week of actigraphy and activity/sleep logs
  - $150 for completing the clinical assessments and fMRI
- Visit 4 - $75 for completing previous week of actigraphy and activity/sleep logs
- Visit 5 - $225
  - $75 for completing previous week of actigraphy and activity/sleep logs
  - $150 for completing clinical assessments and fMRI
- Visit 6 - $75 for completing previous week of actigraphy and activity/sleep logs
- Visit 7 - $400
  - $75 for completing previous week of actigraphy and activity/sleep logs
  - $150 for completing clinical assessments and fMRI
  - $175 bonus for completing the study
- Travel stipend - $80 - $240 for Visit 1-7 (e.g. up to 8 visits to the research clinic including coming to the clinic twice for activities associated with Visit 2)
  - The travel stipend will be $10 - $30 per study visit to account for the varying distance which different participants must travel to the lab. The travel stipend will always be at least $10, increasing by approximately $1 per mile traveled, up to a maximum of $30 for 30 miles or more. Mileage will be rounded up to the nearest whole number to determine the dollar amount given, e.g., 10.4 miles would amount to a travel allowance of $11.
  - Participants will be asked to verify from which location they will be traveling to lab visits during their phone screen and reconfirm this at Lab Visit 1, which will determine their stipend for Visits 1-7.
  - If the fMRI occurs on a different day from the research clinic visit, the travel stipend is only provided for the visit to the clinic (e.g. Rachel Upjohn Building).

## Screen Failures

Criteria for screen failures include:

- Positive urine drug screen at baseline
- Study team learns of study exclusion criteria that arises during the baseline week

# Study Intervention

## Description

Morning light treatment is a non-invasive and low-risk therapeutic option for trauma-related stress and disturbed mood and arousal. The light is transmitted directly from retinal photoreceptors to the amygdala, a key brain region in the manifestation of traumatic stress symptoms. The light is delivered using a pair of Re-Timer glasses that have been adjusted for each participant to optimize the light delivery.

## Administration & Dosing

- Self-administered in own home
- First thing upon waking (or up to 1 hour earlier to accommodate morning commitments like work or childcare)
- Light intensity is about 500 lux
- Duration is pre-set for either 15, 30 or 60 minutes/day depending on study arm
- Daily administration for 4 consecutive weeks (starting the morning after Visit 3)

## Fidelity

- Participants are provided with a pre-set alarm clock to the prescribed wake up time (based on the baseline week of actigraphy); they are instructed to use their own alarm clock as a backup.
- The Re-Timer glasses are configured with a pre-set timer based on prescribed duration. Participants are to activate the timer at the start of each treatment session.
- Participants will record times of use and any interruptions or alterations to the treatment session in the daily sleep/activities log.
- Participants will receive a daily communication asking a Yes/No question completion of light therapy.
- Participants will meet with the study team weekly to review light treatment data (downloaded from the Re-Timer glasses).

## Measures to Minimize Bias

### Randomization

Participants will be randomized at Visit 3, after the week-long baseline period, to one of 3 daily light durations (15, 30 or 60 minutes/day) using a secure web-based treatment assignment system implemented by CSCAR (cascar-randomization.appspot.com). This system uses a minimization approach to reduce imbalances in covariates following each round of treatment assignment by using non-uniform assignment probabilities for the different treatment groups. The following minimization variables will be used: combat vs. non-combat index trauma; DASS; use of psychoactive medication; ages, sex and race.

### Blinding

The outcomes assessor will be blinded to study arm, along with rest of the study team *except for* the PI, study coordinator and designated research assistants. These individuals need to remain unblinded to perform safety assessments and provide feedback on intervention compliance. Blinded staff will wear buttons as an upfront visual cue to remind participants not to talk about their treatment.

## Concomitant Therapy

Patients are allowed to continue their usual care throughout the study. All concomitant treatments must be stable for at least 30 days prior to enrollment, *and* be maintained throughout the study period. **Allowable therapies** include, but are **not limited to**:

- Prescribed psychotropic medications (except for benzodiazepines, SSRIs, SNRIs, beta blockers, and opioids)

## Contraindicated Therapy

Current participation in psychotherapy or other non-pharmacological treatments for trauma stress is an exclusion criterion because of potentially confounding effects on study outcomes.

Likewise, current or new use of benzodiazepines, SSRIs, SNRIs, beta blockers, or opioids is contraindicated because of their potential effects on amygdala reactivity.

# End-of-Intervention/End-of-Study

## Discontinuation of Intervention

Use of the Re-Timer will be stopped in the event of:

- Significant clinical deterioration or expressed suicidal ideation at the determination of the study team
- Severe treatment side effects at the determination of the study team
- Significant decline in physical health making compliance with the intervention either ill-advised or challenging to complete
- Participant request to discontinue therapy

Reasons for discontinuation will be documented, and all outcome assessments will proceed as scheduled, if possible.

## Participant Discontinuation or Withdrawal from Study

Reasons why a participant might be withdrawn from the study:

- Participant request
- Any new diagnosis that would be considered part of the original exclusion criteria for this study
- Positive breathalyzer or drug test at the study at Visits 3, 4, 5 or 6
- Start of new treatment or therapy during study period
- Significant study intervention non-compliance, i.e. inability or unwillingness to complete study intervention or outcome assessments
- Any clinical event or other medical condition or situation such that continued participation in the study would not be in the best interest of the subject
- If the participant meets an exclusion criterion (either newly developed or not previously recognized) any time
- Lost-to-follow up

If possible, reasons for withdrawal will be documented. Data from dropouts or withdraws will be retained to minimize data loss.

# Study Assessments & Procedures

## Eligibility Confirmation

At the screening (initial) visit to the lab, Day 0, participants will complete a number of self-report questionnaires to confirm eligibility including self-report health questionnaires and basic demographics.

## Vision Testing

Participants will undergo a brief vision test that involves standing a set distance from a standard Snellen-type eye chart as well as a screening for color-blindness using a common color vision test that involves looking at a circular pattern comprised of many dots of various colors, brightness and sizes.

## Psychological Assessment

Participants will undergo a clinical-administered assessment to identify lifetime and current psychiatric diagnoses, and complete self-report instruments to assess lifetime occurrence and severity of trauma.

- Structured Clinical Interview for DSM-5 (SCID-5-RV) [57]
- Brief Trauma Questionnaire [76]
- Childhood Trauma Questionnaire [77]

## Sleep Assessment

We will assess sleep using both objective (actigraphy) and subjective (PSQI) measures to use as compliance indices and covariates. Daily logbooks will supplement these assessments, and are designed to capture wake/bed times, treatment times, etc.

- Wrist actigraphy using the Actigraph Spectrum every day for duration of study
- Daily Logs
- Pittsburgh Sleep Quality Index (PSQI) [95]
- Morning-ness & Eveningness (Owl-Lark) Questionnaire [135]
- Insomnia Severity Index (ISI) [136]

## Clinician-administered* & Self-reported Assessments

We will use validated clinician-rated and self-reported measures of current traumatic stress symptoms. Clinician-administered instruments will be video-recorded and used for fidelity checks by MPI, Dr. Zalta. We expect to review approximately 20% (random sample) of the recordings.

- *Posttraumatic Stress Disorder Symptom Scale for DSM-5 (PSSI-5) [79]
- PTSD Checklist for DSM-5 (PCL-5) [80]
- *Hamilton Depression Rating Scale (HAM-D) [81]
- *Hamilton Anxiety Rating Scale (HAM-A) [82]
- Depression Anxiety Stress Scale (DASS) [58]
- Beck Scale for Suicidal Ideation (BSSI) [133]
- Work and Social Adjustment Scale (WSAS) [134]

## Other Self-reported Assessments

- Demographics/health Information/Student Status forms
- fMRI screening form
- Treatment Expectation & Treatment Satisfaction Questionnaires
- Follow up Questionnaire completed at study completion

## fMRI

Amygdala reactivity will be measured using fMRI and change in BOLD signal measures across the study period. Images will be acquired using an Echo-Planar Imaging sequence (TR=800 ms, FOV=23, slice=2.4mm, 96x96 matrix) to measure brain function at rest and during the EFAT task, optimized to reduce susceptibility artifact. fMRIs will be scheduled at the North Campus facility and must occur within 7 days of the clinician-administered and self-reported assessments of trauma stress and mood (see Section 9.3)

### fMRI Practice

As part of the screening process, individuals will be able to lie in the mock scanner located at the Rachel Upjohn Building. This scanner is built to size, and includes simulating scanner noise from speakers so people can hear the noises associated with scanning.

### Resting brain function

Participants are asked to “remain still, keep your eyes open and the fixation mark, let your mind wander freely, but do not fall asleep.”

### Emotional Face Assessment Task (EFAT)

The EFAT is an effective probe of amygdala reactivity [72] that has been validated in (1) anxious patients [73, 74]; (2) in anxious and healthy participants with pharmacological challenges [73, 75]; and (3) in anxious patients undergoing psychotherapy [74]. Participants will view a trio of faces, and are instructed to match one of the two faces across the top of the screen that expresses the same emotion as the target face shown at the bottom. The target and congruent probe face display one of five expressions (angry, happy, fearful, sad, neutral) and the other (incongruent) probe face always displays a neutral (or happy if the target is neutral) expression. This design isolates amygdala reactivity specifically to different types of cues conveyed by each expression which have similar perceptual characteristics except for the emotional signal. The paradigm consists of 20 face trials per expression and 20 shape trials (each 3 seconds in duration), presented in pseudorandomized order, separated by a 3-7 sec inter-trial ‘jitter” (blank screen, fixation cross). During all sessions, our primary measure will be brain response to signals of threat (e.g. fear > happy). Besides fMRI signal, we will also obtain behavioral measures of accuracy and response time to verify participants are following the instructions. To ensure participants understand task instructions, participants will complete practice trials with faces not used in the experiment before undergoing fMRI.

### Emotional Regulation Task

The ERT assesses an individual’s ability to regulate emotions. The task is designed to elicit positive and negative emotional states; individuals view positive (e.g., flowers), negative (e.g., crying person), and neutral (e.g. fire hydrant) images from a curated repository, the International Affective Picture System. Participants are instructed to regulate their emotional response by either increasing the amount of their emotional response, decreasing it, or simply view the image without any purposeful regulation. The testing paradigm consists of nine iterations of 5 conditions: decrease negative, watch negative, increase positive, watch positive, and watch neutral. The participant’s view alternates between a white fixation cross and a picture with the prompt (increase, decrease, or watch) superimposed. During this time the participant tries to regulate their emotion as instructed. Between each trial participants are asked to rate the intensity of the emotion they experienced.

## Treatment expectation & Treatment Satisfaction

Prior to the start of their morning light treatment, participants will rate their expectations for treatment on a 0-10 numerical rating scale (0 “do not expect to benefit” to 10 “expect to benefit a lot”). Participants will then rate their level of satisfaction with their treatment at the final study visit.

## Other Research Procedures

### Urine drug/pregnancy screen

All female participants of childbearing age take a urine pregnancy test at visit 1, and every visit with an fMRI; additionally, we will also do a dip stick drug screen to assess for illicit drug use on all participants during lab visit 1 , lab visit 2, and at every visit with an fMRI.

### Breathalyzer

Participants will be evaluated for recent alcohol use at each study visit by blowing into a breathalyzer.

### Self-reported medical history

Participants will fill out a comprehensive medical history and current medications.

## Safety Assessments

### SAFTEE for treatment side effects

An unblinded study team member will administer the SAFTEE (Systematic Assessment for Treatment Emergent Events) at weekly study visits.

If a participant reports a significant worsening of symptoms as indicated by a score of 4 (“bothered quite a bit”) or 5 (“bothered extremely”), or endorses any of the following trigger points, the study team will contact Dr. Burgess as soon as possible who will then contact the participant for additional information. Decisions on subsequent actions (e.g. withdrawal, referral to treatment, etc.) will be made by the MPIs made decide to withdraw the participant from the study, and refer them to appropriate treatment.

**Trigger Point Items from the SAFTEE**

- Loss of consciousness (2 or more)
- Seizures (2 or more)
- Difficulty swallowing (3 or more)
- Chest pain (2 or more)
- Shortness of breath (2 or more)
- Rapid heart rate (2 or more)
- Irregular heart beat (2 or more)
- Feeling restless or like you cannot stay still (3 or more)
- Too much energy (3 or more)
- Jumpiness or feeling jittery (3 or more)
- Feeling excited (3 or more)
- Overactive (3 or more)
- Elated (3 or more)

### Columbia Suicide Severity Rating Scale (C-SSRS)

The C-SSRS is used widely in a variety of clinical and research settings, and is sensitive to change over time [97, 132]. All instrument administrators will complete the required online clinical trials training module provided by the developers of the measure prior to administering the instrument. Updated training is required every 2 years. Study staff will review the C-SSRS before the participant leaves the session. If a participant reports significant suicidality at any point during the study (C-SSRS item 4 or 5 = yes), Co-I, Dr. Horwitz (a specialist in suicide), will review the intensity of ideation score (range = 2-25) and speak with the participant to discuss the participant’s symptoms and conduct an evaluation before the participant leaves the study session.

# Adverse Events

The Data and Safety Monitoring Plan is the definitive source for this study’s strategy for upholding data integrity and participant safety. Briefly, this study is considered “minimal risk” because the probability and magnitude of harm or discomfort anticipated in the research are not greater than those normally encountered in daily life or during the performance of routine medical and psychological care. Safety of subjects will be ensured by the continued monitoring of their mental health and medical status. Participants will all have access to treatment-as-usual services to address any clinical concerns or study-induced adverse effects.

This study will use an independent safety monitor with experience in conducting clinical trials for psychiatric disorders, fMRI, biostatistics, and human subjects protections and research ethics. NIMH will approve the safety monitor prior to initial of the study.

## Definitions

An adverse event (AE) is any unfavorable or unintended symptom, sign, or disease associated with a medical treatment or procedure that may or may not be related to the treatment or procedure. Adverse events can be related to the treatment or to the disorder being treated (e.g. clinical worsening), as well as to a concurrent disorder or treatment (e.g. depression or its treatment), or they could be entirely unrelated to any of these (e.g., motor vehicle accident).

### Serious adverse event (SAE)

This study will use the FDA definition of SAE:

- Death
- Hospitalization or prolongation of hospitalization, or emergency room visits
- Persistent or significant disability or incapacity
- Suicide plans or attempts

## Classification

All AEs will be classified according to severity and attribution.

### Severity

- (0) No adverse event or within normal limits
- (1) Mild adverse event – did not require treatment
- (2) Moderate adverse event – resolved with treatment
- (3) Severe adverse event – resulted in inability to carry on normal activities *and* required professional medical attention
- (4) Life threatening or disabling adverse event
- (5) Fatal adverse event

### Attribution

- Definite: The AE is clearly related to the investigational agent(s)
- Probable: The AE is likely related to the investigational agent(s)
- Possible: The AE may be related to the investigational agent(s)
- Unlikely: The AE is doubtfully related to investigational agent(s)
- Unrelated: The AE is clearly not related to investigational agents(s)

## Reporting

All SAEs will be reported by the PI to the safety monitor, IRBMED, NIMH, and FDA as appropriate within 5 days of the study team becoming aware of an event. Any initial SAE report will be followed by submission of a completed resolution report. In addition, the PIs will prepare an annual report on data collection and occurrence of any adverse events for review by the above-listed groups.

In the event that a patient withdraws from the study or the investigators decide to discontinue a patient due to SAE, the patient will be monitored by the PIs via ongoing status assessment until:

- A resolution is reached (i.e., the problem has resolved or stabilized with no further changes expected)
- The SAE is determined to be clearly unrelated to the study intervention, or
- The SAE results in death

Should a large number of unexpected SAEs occur, we will modify or terminate the trial if the events are severe. We will also monitor safety alerts, defined as events that are relevant to the study populations and pose safety risks to study participants. Examples of safety alerts would include a clinically significant increase in participant depression or anxiety or certain responses on the SAFTEE. Both SAEs and safety alerts will be tracked using a standardized form recording the date of the event, type of event, attribution of the event (e.g., judgment regarding whether it was intervention related), whether the event was resolved or controlled, and the resolution date.

## Unanticipated Problems

Unanticipated problems, in general, will include any incident, experience, or outcome that meets ALL of the following 3 criteria:

- It is unexpected (in terms of nature, severity, or frequency) given (a) the research procedures that are described in the protocol-related documents, such as the IRB-approved research protocol, informed consent document, or other research materials; and (b) the characteristics of the subject population being studied.
- It is related or possibly related to participation in the research (possibly related means there is a reasonable possibility that the incident, experience, or outcome may have been caused by the procedures involved in the research).
- It suggests that the research places subjects or others at a risk of unknown harm or addition/increased frequency of harms (including physical, psychological, economic, legal, or social harm) than was previously known or recognized.

The MPIs will have ultimate responsibility for event reporting. Monitoring will occur by the research team who will then bring all AEs and UaPs to the attention of the MPIs for evaluation, gradation, and appropriate reporting. The MPIs and the study coordinator will be responsible for overseeing data integrity, safety monitoring, and reporting of adverse events.

# Statistical Considerations

## General Approach

The objective of this study is to establish a signification dose response relationship between the duration of morning light treatment and reduction in amygdala reactivity from baseline to treatment week 2 (study week 3) and/or treatment week 4 (study week 5).

Statistical analyses will include intent-to-treat and completer analysis. Data will be analyzed using responses at all three times (baseline, 2 and 4 weeks) as dependent variables using a mixed-effects longitudinal model [106, 107] with participants as random intercepts to account for between participant variability and time as a predictor.

### Hypothesis

There will be a significant dose response relationship between light duration and reduction in amygdala reactivity.

### Go/No-go Criteria

A meaningful reduction in amygdala reactivity (Cohen’s d≥0.5) must be observed with one of the three daily light durations at treatment week 2 and/or treatment week 4.

## Analysis of Primary Outcome

We will first graphically explore the relationship between responses and dose by time and specify how dose and time will be parameterized. For example, if a linear dose-dependent relationship is shown at each follow-up time (e.g., incremental drop in amygdala reactivity with increasing duration of daily light pulse), we will assess it by including dose (in minutes) and will include the interactions of time by dose to assess how/if the relationship changes over time (e.g., no difference across groups at baseline and dose-dependent relationship at weeks 2 and 4). A test of overall dose response relationship between duration of daily light pulse and reduction from baseline in amygdala reactivity will be established based on the significance of the interaction term. Time can also be included as categorical indicators to see, for example, if 4 weeks, relative to 2 weeks achieve even greater (or no greater) response.

If a nonlinear dose-dependent relationship is shown in graphs, it will be explored using indicators of dose groups. If the relationships at 2 weeks differ from those at 4 weeks, we will analyze them using separate models.

Statistical analysis will be performed using SAS 9.3. Regarding missing data, if “non-ignorable” patterns are detected, then “pattern mixture”, “selection models”, or other appropriate strategies will be used to examine the impact of the missing data pattern on the key outcomes [108, 109]. All analyses will include tests for sex differences, in order to examine sex as a biological variable.

## Exploratory Analyses

### fMRI

In addition to the effect size comparison for the ROI analysis, we will analyze the data with the mixed-effects longitudinal regression model described above. We will also conduct exploratory whole-brain voxel-wise analyses, employing a similar statistical approach across the entire brain in an exploratory search to generate hypotheses for subsequent study. Statistical maps for the whole brain analysis will be created using a threshold of p<0.001 with a cluster threshold of at least 10 voxels. Brain images will be entered into second-level analyses implemented in SPM12 to mirror analyses performed with extracted values. We will also conduct exploratory amygdala analyses to evaluate: 1) different sub-regions within the amygdala; 2) activation outside of amygdala using whole-brain voxel-wise analyses; and 3) using amygdala circuit seeds for functional (psychophysiological interaction analyses) [110], resting state [111, 112] and effective (dynamic causal modeling) connectivity analyses [113].

### Traumatic stress symptoms

We will also examine the clinician-administered (PSSI-5, HAM-D, HAM-A) and self-report measures (DASS, PCL-5) of traumatic stress symptoms with a mixed-effects longitudinal regression model as described above.

### Baseline Analysis and Covariate Adjustment

With minimization randomization, we do not expect groups differences in the distributions of potentially prognostic baseline variables. We will check for imbalances in other baseline variables not used in minimization including childhood trauma and objective measures of total sleep time (sleep duration), wake after sleep onset (sleep continuity) and sleep quality (PSQI) assessed prior to baseline and include them as covariates in the analyses as needed. To explore how much of the change in amygdala reactivity and traumatic symptom reductions are associated with an improvement in sleep, we will also include separately each potential intermediate outcomes of the weekly average of total sleep time, wake after sleep onset and sleep quality assessed prior to each study visit (Days 22, 36) as predictors of outcomes. We will also explore if diagnostic status (baseline SCID) moderates the treatment response.

# Supporting Documentation & Oversight Considerations

## Regulatory, Ethical & Study Oversight

### Informed Consent Process

The recruitment and informed consent process is designed to provide information about study participation multiple times and in different formats. Individuals expressing interest will be provided with a verbal explanation of study procedures and the risk-benefit profile and/or be able to read basic study information on the umhealthresearch.org website. From there, individuals are provided with an IRB-approved copy of the consent document to review at their leisure.

The informed consent dialog will take place at the initial study visit (Day 0). It will consist of a dialog between the study team and the potential participant. The discussion will focus on the voluntary nature of research, expectations for participation, a discussion of the risks and benefits associated with being in the study, the difference between research and clinical care, and include time for questions.

The physical location for the consent dialog will be private research space within the Sleep and Circadian Research Laboratory.

## Data Retention

Research data will be retained for both study record-keeping purposes and for future unspecified research use according to IRBMED guidelines for federally-funded projects. All paper and computer records will be identified by the subject ID code rather than name or other identifier. Consent documents and other forms with identifiers will be stored in a separate double-locked setting.

Data from participants who withdraw from the study will be retained in the study database.

### Study-record keeping

Hard copies of the data will be maintained for 7 years after which they will be destroyed. Files will be stored in double-locked environment and access-limited under the purview of the MPIs. All research records will be identified by subject ID; pre-screening records will be scrubbed of identifiers and date shifted to preserve confidentiality.

Study database records will be identified by subject ID, and the link between identifiers and subject ID will be maintained by the MPI, Dr. Burgess, and lab manager. The link will be destroyed after submission of the NIH final report for Phase 2 (R33) if funded, or publication of the primary manuscript for Phase 2, whichever is later. If Phase 2 is not funded, then the link will be destroyed after submission of the final NIH report for this study (Phase 1 – R61).

#### Protected Health Information (PHI)

We will abstract basic eligibility and contact information from the Michigan Medicine electronic medical records system, MiChart. These data will be stored separately from study data and will be retained with identifiers throughout the duration of the study. Once the study concludes, the identifiers will be destroyed.

### Data Access

Study data will be stored using the University of Michigan instance of REDCap with access restricted based on study roles and responsibilities. The video files will be stored on MBox. The MPI on the study, Dr. Zalta of University of California at Irvine, will have access rights to all data elements.

### Data for future unspecified research use

De-identified study databases will be locked and archived for future unspecified research. These data will be used for analyses related to the main study, new analyses, and in grant proposals for new research.

- The data will be stored on Michigan Medicine servers in REDCap and/or statistical datasets that are stripped of identifiers
- Access to data will be at the discretion of the MPIs
- Any data shared with collaborators will shared using secure data transfer methods (e.g. MiShare)

## Safety Oversight

### Clinical Monitoring

The clinical status of participants is monitored throughout the study. Any >25% worsening from baseline measures of depression and anxiety are flagged for review by the MPIs. Any >50% worsening of these measures is grounds for discontinuing the participant, with careful follow up (no participant in our pilot study worsened to this degree).

- MPI, Dr. Zalta is the study expert in PTSD and traumatic stress. She will have weekly clinical coordinating meetings with Co-Is Dr. Horwitz and Ms. Mooney regarding the clinician-administered assessments, and review the self-report instruments as needed. She, and Dr. Horwitz and Ms. Mooney, will be available to consult on all noted psychological events. Any indication of clinical worsening or suicidal ideation will be managed in real-time before the participant leaves the lab. In cases where additional treatment is recommended or indicated, appropriate referrals or transport to UM Psychiatric Emergency Department will be provided.
- Co-I, Dr. Abelson, will be available to consult on all medical matters and SAFTEE reports. As with instances of worsening psychological status, treatment referrals and communication with personal physicians will be provided as needed.
- Co-Is Dr. Duval and Abelson will be available to consult on abnormal fMRI findings. Any incidental findings will be discussed with the participant and their personal physician with referrals made as needed.
- Adjudication on whether to withdraw a participant from the study because of change in clinical status will be the purview of the MPIs and Co-Is.

### Independent Safety Monitor

Dr. Adrian Preda (medical doctor) of University of California at Irvine, Professor of Clinical Psychiatry, will serve as the independent safety monitor of the study. He will review adverse events, and offer guidance regarding early stopping rules.

Contact Information for Dr. Preda:

Email: [apreda@uci.edu](mailto:apreda@uci.edu)

Office telephone: 714-456-8688

### Quality Assurance

The study will use the Michigan Medicine instance of REDCap, a web-based electronic data capture system that is secure and HIPAA-compliant. Access is restricted by role within the study database, and under the purview of the MPI, Dr. Burgess. Data validation and data quality rules will be implemented to minimize errors in data capture, entry and analysis.

REDCap provides automated export procedures to common statistical packages in which automated syntax can be used to score questionnaire responses thereby reducing errors associated with hand calculations or Excel formulas. It is standard procedure in the Burgess Lab that all data manipulations are double-checked and initialed by a second study team member.

Weekly team meetings in which data are reviewed will further serve as a quality assurance measure. Data reviewed will include information about mental and physical health symptoms of participants, and various psychological characteristics gleaned from the questionnaire battery and participant report.

#### Video Recordings of Clinician Assessments

All clinician-administered assessments will be videotaped with a random sample of ~20% reviewed for reliability and validity checks. Recordings will be stored on MBox, and be accessible only to authorized study team members. Video files will be available to MPI, Dr. Zalta for review via her validated UMICH access or MiShare, depending on the storage location.

## Abbreviations

AE – Adverse event

BOLD – Blood oxygen dependent level

C-SSRS – Columbia Suicide Severity Rating Scale

DASS – Depression Anxiety Stress Scales

DSM-5 – Diagnostic & Statistical Manual of Mental Disorders

EFAT – Emotional Faces Assessment Task

ERT – Emotional Regulation Task

fMRI – Functional magnetic resonance imaging/images

HAM-A – Hamilton Anxiety Scale

HAM-D – Hamilton Depression Scale

HIPAA – Health Insurance Portability & Accountability Act

ipRGC – Intrinsically photosensitive retinal ganglion cells

ISI – Insomnia Severity Index

MEQ – Morningness-Eveningness (Owl-Lark) Questionnaire

MICHR – Michigan Institute for Clinical Health Research

MPI – Multiple Principal Investigators

NIH – National Institutes of Health

NIMH – National Institute of Mental Health

PCL-5 – PTSD Checklist for DSM-5

PHI – Protected Health Information

PSQI – Pittsburgh Sleep Quality Index

PSSI-5 – Posttraumatic Stress Disorder Symptom Scale

PTSD – Posttraumatic stress disorder

ROI – Region of interest

SAE – Serious adverse event

SAFTEE – Systematic Assessment for Treatment Emergent Events

SCID-5-RV – Structured Clinical Interview

SSRI – Selective serotonin re-uptake inhibitor

UaP – Unanticipated problem

WSAS – Work and Social Adjustment Scale

WOC – Without compensation

# References

1. Gooley JJ, Lu J, Chou TC, Scammell TE, Saper CB. Melanopsin in cells of origin of the retinohypothalamic tract. Nature Neuroscience. 2001;4(12):1165.

2. LeGates TA, Fernandez DC, Hattar S. Light as a central modulator of circadian rhythms, sleep and affect. Nat Rev Neurosci. 2014;15(7):443-54.

3. Lovato N, Lack L. Circadian phase delay using the newly developed Re-timer portable light device. Sleep Biol Rhythms. 2016;14:157-64.

4. Wright HR, Lack LC, Kennaway DJ. Differential effects of light wavelength in phase advancing the melatonin rhythm. J. Pineal Res. 2004;36(2):140-4.

5. Wright HR, Lack LC, Partridge KJ. Light emitting diodes can be used to phase delay the melatonin rhythm. J. Pineal Res. 2001;31(4):350-55.

6. Zeitzer JM, Dijk DJ, Kronauer RE, Brown EN, Czeisler CA. Sensitivity of the human circadian pacemaker to nocturnal light: melatonin phase resetting and suppression. J Physiol. 2000;526.3:695-702.

7. Eastman CI, Young MA, Fogg LF, Liu L, Meaden PM. Bright light treatment of winter depression: A placebo-controlled trial. Arch Gen Psychiatry. 1998;55:883-89.

8. Burgess HJ, Fogg LF, Young MA, Eastman CI. Bright light therapy for winter depression - Is phase advancing beneficial? Chronobiol Int. 2004;21:759-75.

9. Vandewalle G, Maquet P, Dijk DJ. Light as a modulator of cognitive brain function. Trends Cogn Sci. 2009;13(10):429-38.

10. Fisher PM, Madsen MK, Mc Mahon B, Holst KK, Andersen SB, Laursen HR, Hasholt LF, Siebner HR, Knudsen GM. Three-week bright-light intervention has dose-related effects on threat-related corticolimbic reactivity and functional coupling. Biol Psychiatry. 2014;76(4):332-9.

11. Rauch SL, Whalen PJ, Shin LM, McInerney SC, Macklin ML, Lasko NB, Orr SP, Pitman RK. Exaggerated amygdala response to masked facial stimuli in posttraumatic stress disorder: a functional MRI study. Biol Psychiatry. 2000;47(9):769-76.

12. Hayes JP, Hayes SM, Mikedis AM. Quantitative meta-analysis of neural activity in posttraumatic stress disorder. Biol Mood Anxiety Disord. 2012;2:9.

13. Sheline YI, Barch DM, Donnelly JM, Ollinger JM, Snyder AZ, Mintun MA. Increased amygdala response to masked emotional faces in depressed subjects resolves with antidepressant treatment: an fMRI study. Biol Psychiatry. 2001;50(9):651-8.

14. Phan KL, Fitzgerald DA, Nathan PJ, Tancer ME. Association between amygdala hyperactivity to harsh faces and severity of social anxiety in generalized social phobia. Biol Psychiatry. 2006;59(5):424-9.

15. Felmingham K, Kemp A, Williams L, Das P, Hughes G, Peduto A,Bryant R. Changes in anterior cingulate and amygdala after cognitive behavior therapy of posttraumatic stress disorder. Psychol Sci. 2007;18(2):127-9.

16. Peres JF, Newberg AB, Mercante JP, Simao M, Albuquerque VE, Peres MJ, Nasello AG. Cerebral blood flow changes during retrieval of traumatic memories before and after psychotherapy: a SPECT study. Psychol Med. 2007;37(10):1481-91.

17. Martensson B, Pettersson A, Berglund L,Ekselius L. Bright white light therapy in depression: A critical review of the evidence. J Affect Disord. 2015;182:1-7.

18. Al-Karawi D, Jubair L. Bright light therapy for nonseasonal depression: Meta-analysis of clinical trials. J Affect Disord. 2016;198:64-71.

19. Langevin JP, De Salles AA, Kosoyan HP, Krahl SE. Deep brain stimulation of the amygdala alleviates post-traumatic stress disorder symptoms in a rat model. J Psychiatr Res. 2010;44(16):1241-5.

20. Stidd DA, Vogelsang K, Krahl SE, Langevin JP, Fellous JM. Amygdala deep brain stimulation is superior to paroxetine treatment in a rat model of posttraumatic stress disorder. Brain Stimul. 2013;6(6):837-44.

21. Langevin JP, Koek RJ, Schwartz HN, Chen JWY, Sultzer DL, Mandelkern MA, Kulick AD, Krahl SE. Deep brain stimulation of the basolateral amygdala for treatment-refractory posttraumatic stress disorder. Biol Psychiatry. 2016;79(10):e82-e84.

22. Burgess HJ, Rizvydeen MR, Kimura M, Pollack MH, Hobfoll SE, Rajan KB, Burns JW. An open trial of morning bright light treatment among US military veterans with chronic low back pain: A pilot study. Pain Med. In press.

23. Perkonigg A, Kessler RC, Storz S, Wittchen HU. Traumatic events and post-traumatic stress disorder in the community: prevalence, risk factors and comorbidity. Acta Psychiatr Scand. 2000;101(1):46-59.

24. Stein DJ, Chiu WT, Hwang I, Kessler RC, Sampson N, Alonso J, Borges G, Bromet E, Bruffaerts R, de Girolamo G, et al. Cross-national analysis of the associations between traumatic events and suicidal behavior: findings from the WHO World Mental Health Surveys. PLoS One. 2010;5(5):e10574.

25. Wang PS, Berglund P, Olfson M, Pincus HA, Wells KB, Kessler RC. Failure and delay in initial treatment contact after first onset of mental disorders in the National Comorbidity Survey Replication. Arch Gen Psychiatry. 2005;62(6):603-13.

26. Watts BV, Shiner B, Zubkoff L, Carpenter-Song E, Ronconi JM, Coldwell CM. Implementation of evidence-based psychotherapies for posttraumatic stress disorder in VA specialty clinics. Psychiatr Serv. 2014;65(5):648-53.

27. Hoge CW, Grossman SH, Auchterlonie JL, Riviere LA, Milliken CS, Wilk JE. PTSD treatment for soldiers after combat deployment: low utilization of mental health care and reasons for dropout. Psychiatr Serv. 2014;65(8):997-1004.

28. Najavits LM. The problem of dropout from "gold standard" PTSD therapies. F1000Prime Rep. 2015;7:43.

29. Kantor V, Knefel M, Lueger-Schuster B. Perceived barriers and facilitators of mental health service utilization in adult trauma survivors: A systematic review. Clin Psychol Rev. 2017;52:52-68.

30. Bradley R, Greene J, Russ E, Dutra L, Westen D. A multidimensional meta-analysis of psychotherapy for PTSD. Am J Psychiatry. 2005;162(2):214-27.

31. Watts BV, Schnurr PP, Mayo L, Young-Xu Y, Weeks WB, Friedman MJ. Meta-analysis of the efficacy of treatments for posttraumatic stress disorder. J Clin Psychiatry. 2013;74(6):e541-50.

32. Stein DJ, Ipser J, McAnda N. Pharmacotherapy of posttraumatic stress disorder: a review of metaanalyses and treatment guidelines. CNS Spectr. 2009;14(1 Suppl 1):25-31.

33. Morin LP. Neuroanatomy of the extended circadian rhythm system. Exp Neurol. 2013;243:4-20.

34. Baxendale S, O'Sullivan J, Heaney D. Bright light therapy for symptoms of anxiety and depression in focal epilepsy: randomised controlled trial. Br J Psychiatry. 2013;202(5):352-6.

35. Youngstedt SD, Kline CE, Ginsberg JP, Zielinski MR, Hardin JW. Bright light treatment for high-anxious young adults: a randomized controlled pilot study. Depress Anxiety. 2011;28(4):324-32.

36. Youngstedt SD,Kripke DF. Does bright light have an anxiolytic effect? - an open trial. BMC Psychiatry. 2007;7:62.

37. Kessler RC. Posttraumatic stress disorder: the burden to the individual and to society. J Clin Psychiatry. 2000;61 Suppl 5(4-12); discussion 13-4.

38. Kilpatrick DG, Resnick HS, Milanak ME, Miller MW, Keyes KM, Friedman MJ. National estimates of exposure to traumatic events and PTSD prevalence using DSM-IV and DSM-5 criteria. J Trauma Stress. 2013;26(5):537-47.

39. Bremner JD. Traumatic stress: effects on the brain. Dialogues Clin Neurosci. 2006;8(4):445-61.

40. Shin LM. The amygdala in post-traumatic stress disorder. In Post-Traumatic Stress Disorder: Basic Science and Clinical Practice. Shiromani P, Keane T, and LeDoux JE, Editors. 2009. Humana Press. p. 319-44.

41. LeDoux JE. Emotion circuits in the brain. Annu Rev Neurosci. 2000;23:155-84.

42. Rauch SL, van der Kolk BA, Fisler RE, Alpert NM, Orr SP, Savage CR, Fischman AJ, Jenike MA, Pitman RK. A symptom provocation study of posttraumatic stress disorder using positron emission tomography and script-driven imagery. Arch Gen Psychiatry. 1996;53(5):380-7.

43. Shin LM, Orr SP, Carson MA, Rauch SL, Macklin ML, Lasko NB, Peters PM, Metzger LJ, Dougherty DD, Cannistraro PA, et al. Regional cerebral blood flow in the amygdala and medial prefrontal cortex during traumatic imagery in male and female Vietnam veterans with PTSD. Arch Gen Psychiatry. 2004;61(2):168-76.

44. Phan KL, Coccaro EF, Angstadt M, Kreger KJ, Mayberg HS, Liberzon I, Stein MB. Corticolimbic brain reactivity to social signals of threat before and after sertraline treatment in generalized social phobia. Biol Psychiatry. 2013;73(4):329-36.

45. Pail G, Huf W, Pjrek E, Winkler D, Willeit M, Praschak-Rieder N, Kasper S. Bright-light therapy in the treatment of mood disorders. Neuropsychobiology. 2011;64(3):152-62.

46. Terman M, Terman JS. Light therapy for seasonal and nonseasonal depression: Efficacy, protocol, safety, and side effects. CNS Spectrums. 2005;10:647-63.

47. Gallin PF, Terman M, Reme CE, Rafferty B, Terman JS, Burde RM. Ophthalmologic examination of patients with seasonal affective disorder, before and after bright light therapy. Am J Ophthalmol. 1995;119:202-10.

48. Berson DM. Strange vision: ganglion cells as circadian photoreceptors. Trends Neurosci. 003;26:314-20.

49. Hannibal J, Hindersson P, Ostergaard J, Georg B, Heegaard S, Larsen PJ, Fahrenkrug J. Melanopsin is expressed in PACAP-containing retinal ganglion cells of the human retinohypothalamic tract. Invest Ophthalmol Vis Sci. 2004;45:4202-09.

50. Weng S, Estevez ME, Berson DM. Mouse ganglion-cell photoreceptors are driven by the most sensitive rod pathway and by both types of cones. PLoS One. 2013;8(6):e66480.

51. Dacey DM, Liao HW, Peterson BB, Robinson FR, Smith VC, Pokorny J, Yau KW,Gamlin PD. Melanopsin-expressing ganglion cells in primate retina signal colour and irradiance and project to the LGN. Nature. 2005;433:749-54.

52. Monson CM, Gradus JL, Young-Xu Y, Schnurr PP, Price JL, Schumm JA. Change in posttraumatic stress disorder symptoms: do clinicians and patients agree? Psychol Assess. 2008;20(2):131-8.

53. Liberzon I, King AP, Britton JC, Phan KL, Abelson JL, Taylor SF. Paralimbic and medial prefrontal cortical involvement in neuroendocrine responses to traumatic stimuli. Am J Psychiatry. 2007;164(8):1250-8.

54. King AP, Abelson JL, Britton JC, Phan KL, Taylor SF, Liberzon I. Medial prefrontal cortex and right insula activity predict plasma ACTH response to trauma recall. Neuroimage. 2009;47(3):872-80.

55. Klumpp H, Ho SS, Taylor SF, Phan KL, Abelson JL, Liberzon I. Trait anxiety modulates anterior cingulate activation to threat interference. Depress Anxiety. 2011;28(3):194-201.

56. Rabinak CA, Angstadt M, Sripada CS, Abelson JL, Liberzon I, Milad MR, Phan KL. Cannabinoid facilitation of fear extinction memory recall in humans. Neuropharmacology. 2013;64:396-402.

57. First M, Williams J, Karg R, Spitzer R. Structured Clinical Interview for DSM-5 - Research Version (SCID-5 for DSM-5, SCID-5-RV). 2015, Arlington, VA.: American Psychiatric Association.

58. Lovibond PF,Lovibond SH. The structure of negative emotional states: comparison of the Depression Anxiety Stress Scales (DASS) with the Beck Depression and Anxiety Inventories. Behav Res Ther. 1995;33(3):335-43.

59. Gorka SM, Lieberman L, Klumpp H, Kinney KL, Kennedy AE, Ajilore O, Francis J, Duffecy J, Craske MG, Nathan J, et al. Reactivity to unpredictable threat as a treatment target for fear-based anxiety disorders. Psychol Med. 2017;47(14):2450-60.

60. Gorka SM, Burkhouse KL, Afshar K, Phan KL. Error-related brain activity and internalizing disorder symptom dimensions in depression and anxiety. Depress Anxiety. 2017;34(11):985-95.

61. Burkhouse KL, Gorka SM, Afshar K, Phan KL. Neural reactivity to reward and internalizing symptom dimensions. J Affect Disord. 2017;217:73-79.

62. Weale RA. Age and the transmittance of the human crystalline lens. J Physiol. 1988;395:577-87.

63. Netzer NC, Stoohs RA, Netzer CM, Clark K, Strohl KP. Using the Berlin Questionnaire to identify patients at risk for the sleep apnea syndrome. Ann Intern Med. 1999;131(7):485-91.

64. Hening WA, Allen RP. Restless legs syndrome (RLS): the continuing development of diagnostic standards and severity measures. Sleep Med. 2003;4(2):95-7.

65. Lieverse R, Van Someren EJ, Nielen MM, Uitdehaag BM, Smit JH, Hoogendijk WJ. Bright light treatment in elderly patients with nonseasonal major depressive disorder: a randomized placebo-controlled trial. Arch Gen Psychiatry. 2011;68(1):61-70.

66. Endo A, Nagatani F, Hamada C, Yoshimura I. Minimization method for balancing continuous prognostic variables between treatment and control groups using Kullback-Leibler divergence. Contemp Clin Trials. 2006;27(5):420-31.

67. Kalish LA,Begg CB. Treatment allocation methods in clinical trials: a review. Stat Med. 1985;4(2):129-44.

68. Lin Y, Zhu M, Su Z. The pursuit of balance: An overview of covariate-adaptive randomization techniques in clinical trials. Contemp Clin Trials. 2015;45(Pt A):21-5.

69. Pocock SJ, Simon R. Sequential treatment assignment with balancing for prognostic factors in the controlled clinical trial. Biometrics. 1975;31(1):103-15.

70. Xiao L, Huang Q, Yank V, Ma J. An easily accessible Web-based minimization random allocation system for clinical trials. J Med Internet Res. 2013;15(7):e139.

71. Chang AM, Santhi N, St Hilaire MA, Gronfier C, Bradstreet DS, Duffy JF, Lockley SW, Kronauer RE, Czeisler CA. Human responses to bright light of different durations. J Physiol. 2012;590(Pt 13):3103-12.

72. Hariri AR, Tessitore A, Mattay VS, Fera F, Weinberger DR. The amygdala response to emotional stimuli: a comparison of faces and scenes. Neuroimage. 2002;17(1):317-23.

73. Phan KL, Angstadt M, Golden J, Onyewuenyi I, Popovska A, de Wit H. Cannabinoid modulation of amygdala reactivity to social signals of threat in humans. J Neurosci. 2008;28(10):2313-9.

74. Klumpp H, Fitzgerald DA, Phan KL. Neural predictors and mechanisms of cognitive behavioral therapy on threat processing in social anxiety disorder. Prog Neuropsychopharmacol Biol Psychiatry. 2013;45:83-91.

75. Labuschagne I, Phan KL, Wood A, Angstadt M, Chua P, Heinrichs M, Stout JC, Nathan PJ. Oxytocin attenuates amygdala reactivity to fear in generalized social anxiety disorder. Neuropsychopharmacology. 2010;35(12):2403-13.

76. Schnurr P, Vielhauer M, Weathers F, Findler M, The Brief Trauma Questionnaire (BTQ) Measurement instrument]. 1999, Available from http://www.ptsd.va.gov.

77. Bernstein DP, Stein JA, Newcomb MD, Walker E, Pogge D, Ahluvalia T, Stokes J, Handelsman L, Medrano M, Desmond D, et al. Development and validation of a brief screening version of the Childhood Trauma Questionnaire. Child Abuse Negl. 2003;27(2):169-90.

78. Jonas DE, Cusack K, Forneris CA, Wilkins TM, Sonis J, Middleton JC, Feltner C, Meredith D, Cavanaugh J, Brownley KA, et al. In Psychological and Pharmacological Treatments for Adults With Posttraumatic Stress Disorder (PTSD). 2013: Rockville (MD).

79. Foa EB, McLean CP, Zang Y, Zhong J, Rauch S, Porter K, Knowles K, Powers MB, Kauffman BY. Psychometric properties of the Posttraumatic Stress Disorder Symptom Scale Interview for DSM-5 (PSSI-5). Psychol Assess. 2016;28(10):1159-65.

80. Weathers FW, Litz BT, Keane TM, Palmieri PA, Marx BP,Schnurr PP. The PTSD Checklist for DSM-5 (PCL-5). Scale available from the National Center for PTSD at www.ptsd.va.gov. 2013.

81. Hamilton M. A rating scale for depression. J Neurol Neurosurg Psychiatry. 1960;23:56-62.

82. Hamilton M. The assessment of anxiety states by rating. Br J Med Psychol. 1959;32(1):50-5.

83. Sinha SS. Trauma-induced insomnia: A novel model for trauma and sleep research. Sleep Med Rev. 2016;25:74-83.

84. van Maanen A, Meijer AM, Van der Heijden KB, Oort FJ. The effects of light therapy on sleep problems: A systematic review and meta-analysis. Sleep Med Rev. 2015;29:52-62.

85. Saper CB, Fuller PM. Wake-sleep circuitry: an overview. Curr Opin Neurobiol. 2017;44:186-92.

86. Klumpp H, Roberts J, Kapella MC, Kennedy AE, Kumar A, Phan KL. Subjective and objective sleep quality modulate emotion regulatory brain function in anxiety and depression. Depress Anxiety. 2017;34(7):651-60.

87. Buysse DJ, Ancoli-Israel S, Edinger JD, Lichstein KL,Morin CM. Recommendations for a standard research assessment of insomnia. Sleep. 2006;29(9):1155-73.

88. de Souza L, Benedito-Silva AA, Pires MLN, Poyares D, Tufik S, Calil HM. Further validation of actigraphy for sleep studies. Sleep. 2003;26:81-85.

89. Kushida CA, Chang A, Gadkary C, Guilleminault C, Carrillo O, Dement WC. Comparison of actigraphic, polysomnographic, and subjective assessment of sleep parameters in sleep-disordered patients. Sleep Med. 2001;2:389-96.

90. Burgess HJ, Revell VL, Molina TA, Eastman CI. Human phase response curves to three days of daily melatonin: 0.5 mg versus 3.0 mg. J Clin Endo Metab. 2010;95(7):3325-31.

91. Burgess HJ. Partial sleep deprivation reduces phase advances to light in humans. J Biol Rhythms. 2010;25(6):460-68.

92. Burgess HJ, Eastman CI. Short nights reduce light-induced circadian phase delays in humans. Sleep. 2006;29(1):25-30.

93. Burgess HJ, Eastman CI. Early versus late bedtimes phase shift the human dim light melatonin rhythm despite a fixed morning lights on time. Neurosci Lett. 2004;356:115-18.

94. Burgess HJ,Eastman CI. A late wake time phase delays the human dim light melatonin rhythm. Neurosci Lett. 2006;395:191-95.

95. Buysse DJ, Reynolds CF, Monk TH, Berman SR,Kupfer DJ. The Pittsburgh Sleep Quality Index: A new instrument for psychiatric practice and research. Psychiatry Res. 1989;28:193-213.

96. Dauphinais DR, Rosenthal JZ, Terman M, DiFebo HM, Tuggle C, Rosenthal NE. Controlled trial of safety and efficacy of bright light therapy vs. negative air ions in patients with bipolar depression. Psychiatry Res. 2012;196(1):57-61.

97. Posner K, Brown GK, Stanley B, Brent DA, Yershova KV, Oquendo MA, Currier GW, Melvin GA, Greenhill L, Shen S, et al. The Columbia-Suicide Severity Rating Scale: initial validity and internal consistency findings from three multisite studies with adolescents and adults. Am J Psychiatry. 2011;168(12):1266-77.

98. Friston KJ, Fletcher P, Josephs O, Holmes A, Rugg MD, Turner R. Event-related fMRI: characterizing differential responses. Neuroimage. 1998;7(1):30-40.

99. Friston KJ, Frith CD, Frackowiak RS, Turner R. Characterizing dynamic brain responses with fMRI: a multivariate approach. Neuroimage. 1995;2(2):166-72.

100. Friston KJ, Holmes AP, Poline JB, Grasby PJ, Williams SC, Frackowiak RS, Turner R. Analysis of fMRI time-series revisited. Neuroimage. 1995;2(1):45-53.

101. Josephs O, Turner R, Friston K. Event-related fMRI. Hum Brain Mapp. 1997;5(4):243-8.

102. Rabinak CA, Sripada CS, Angstadt M, de Wit H, Phan KL. Cannabinoid modulation of subgenual anterior cingulate cortex activation during experience of negative affect. J Neural Transm. 2012;119(6):701-7.

103. Rabinak CA, Angstadt M, Lyons M, Mori S, Milad MR, Liberzon I, Phan KL. Cannabinoid modulation of prefrontal-limbic activation during fear extinction learning and recall in humans. Neurobiol Learn Mem. 2014;113:125-34.

104. Walter B, Blecker C, Kirsch P, Sammer G, Schienle A, Stark R, Vaitl D. MARINA: An easy to use tool for the creation of MAsks for Region of INterest Analyses. In 9th International Conference on Functional Mapping of the Human Brain. 2003, Available on CD-Rom in NeuroImage, Vol 19 (2).

105. Tzourio-Mazoyer N, Landeau B, Papathanassiou D, Crivello F, Etard O, Delcroix N, Mazoyer B, Joliot M. Automated anatomical labeling of activations in SPM using a macroscopic anatomical parcellation of the MNI MRI single-subject brain. Neuroimage. 2002;15(1):273-89.

106. Laird NM,Ware JH. Random-effects models for longitudinal data. Biometrics. 1982;38(4):963-74.

107. Hedeker D,Gibbons RD. Longitudinal Data Analysis. 2006, New York: Wiley.

108. Rubin DB. Multiple Imputation for Nonresponse in Surveys. 1987, New York: John Wiley and Son.

109. Schafer JL, Graham JW. Missing data: our view of the state of the art. Psychol Methods. 2002;7(2):147-77.

110. Friston KJ, Buechel C, Fink GR, Morris J, Rolls E, Dolan RJ. Psychophysiological and modulatory interactions in neuroimaging. Neuroimage. 1997;6(3):218-29.

111. Power JD, Cohen AL, Nelson SM, Wig GS, Barnes KA, Church JA, Vogel AC, Laumann TO, Miezin FM, Schlaggar BL, et al. Functional network organization of the human brain. Neuron. 2011;72(4):665-78.

112. Mitra A, Raichle ME. How networks communicate: propagation patterns in spontaneous brain activity. Philos Trans R Soc Lond B Biol Sci. 2016;371(1705):

113. Buxton RB, Uludag K, Dubowitz DJ, Liu TT. Modeling the hemodynamic response to brain activation. Neuroimage. 2004;23 Suppl 1:S220-33.

114. Michalak EE, Murray G, Wilkinson C, Dowrick C, Lam RW. A pilot study of adherence with light treatment for seasonal affective disorder. Psychiatry Res. 2007;149(1-3):315-20.

115. Friedman L, Zeitzer JM, Kushida C, Zhdanova I, Noda A, Lee T, Schneider B, Guilleminault C, Sheikh J, Yesavage JA. Scheduled bright light for treatment of insomnia in older adults. J Am Geriatr Soc. 2009;57(3):441-52.

116. Godlewska BR, Norbury R, Selvaraj S, Cowen PJ, Harmer CJ. Short-term SSRI treatment normalizes amygdala hyperactivity in depressed patients. Psychol Med. 2012;42(12):2609-17.

117. Wulff K, Gatti S, Wettstein JG, Foster RG. Sleep and circadian rhythm disruption in psychiatric and neurodegenerative disease. Nat Rev Neurosci. 11(8):589-99.

118. Johnstone T, Somerville LH, Alexander AL, Oakes TR, Davidson RJ, Kalin NH, Whalen PJ. Stability of amygdala BOLD response to fearful faces over multiple scan sessions. Neuroimage. 2005;25(4):1112-23.

119. Manuck SB, Brown SM, Forbes EE, Hariri AR. Temporal stability of individual differences in amygdala reactivity. Am J Psychiatry. 2007;164(10):1613-4.

120. Tolin DF, Foa EB. Sex differences in trauma and posttraumatic stress disorder: a quantitative review of 25 years of research. Psychol Bull. 2006;132(6):959-92.

121. Kessler RC, Berglund P, Demler O, Jin R, Merikangas KR, Walters EE. Lifetime prevalence and age-ofonset distributions of DSM-IV disorders in the National Comorbidity Survey Replication. Arch Gen Psychiatry. 2005;62(6):593-602.

122. Kessler RC, Demler O, Frank RG, Olfson M, Pincus HA, Walters EE, Wang P, Wells KB, Zaslavsky AM. Prevalence and treatment of mental disorders, 1990 to 2003. N Engl J Med. 2005;352(24):2515-23.

123. Wang PS, Lane M, Olfson M, Pincus HA, Wells KB, Kessler RC. Twelve-month use of mental health services in the United States: results from the National Comorbidity Survey Replication. Arch Gen Psychiatry. 2005;62(6):629-40.

124. Touitou Y. Adolescent sleep misalignment: a chronic jet lag and a matter of public health. J Physiol Paris. 2013;107(4):323-6.

125. Hemenover SH. The good, the bad, and the healthy: impacts of emotional disclosure of trauma on resilient self-concept and psychological distress. Pers Soc Psychol Bull. 2003;29(10):1236-44.

126. Becker-Blease KA, Freyd JJ. Research participants telling the truth about their lives: the ethics of asking and not asking about abuse. Am Psychol. 2006;61(3):218-26.

127. Golden RN, Gaynes BN, Ekstrom RD, Hamer RM, Jacobsen FM, Suppes T, Wisner KL, Nemeroff CB. The efficacy of light therapy in the treatment of mood disorders: A review and meta-analysis of the evidence. Am J Psychiatry. 2005;162:656-62.

128. Rosenthal NE. Diagnosis and treatment of seasonal affective disorder. JAMA. 1993;270:2717-20.

129. Lewy AJ, Bauer VK, Cutler NL, Sack RL, Ahmed S, Thomas KH, Blood ML, Jackson JML. Morning vs evening light treatment of patients with winter depression. Arch Gen Psychiatry. 1998;55:890-96.

130. Auger RR, Burgess HJ, Emens JS, Deriy LV, Thomas SM, Sharkey KM. Clinical Practice Guideline for the Treatment of Intrinsic Circadian Rhythm Sleep-Wake Disorders: Advanced Sleep-Wake Phase Disorder (ASWPD), Delayed Sleep-Wake Phase Disorder (DSWPD), Non-24-Hour Sleep-Wake Rhythm Disorder (N24SWD), and Irregular Sleep-Wake Rhythm Disorder (ISWRD). An Update for 2015: An American Academy of Sleep Medicine Clinical Practice Guideline. J Clin Sleep Med. 2015;11(10):1199-236.

131. Reid KJ, Burgess HJ. Circadian rhythm sleep disorders. Primary care: Clinics in Office Practice. 2005;32:449-73.

132. Mundt JC, Greist JH, Jefferson JW, Federico M, Mann JJ, Posner K. Prediction of suicidal behavior in clinical research by lifetime suicidal ideation and behavior ascertained by the electronic Columbia-Suicide Severity Rating Scale. J Clin Psychiatry. 2013;74(9):887-93.

133. Beck AT, Kovacs M, Weissman A. Assessment of Suicidal Ideation: The Scale for Suicide Ideation. J Consult Clin Psychol. 1979;47(2):343-352.

134. Mundt JC, Marks IM, Shear MK, Greist JM. The Work and Social Adjustment Scale: a simple measure of impairment in functioning. Br J Psychiatry. 2002;180(5):461-464.

135. Horne JA and Ostberg O. A self-assessment questionnaire to determine morningness-eveningness in human circadian rhythms. Int J Chronobiol. 1976; 4(2):97-110

136. Bastien CH, et al. Validation of the Insomnia Severity Index as an outcome measure for insomnia research. Sleep Med. 2001; 2:297-307.
